# Development of a conceptual model and patient-reported outcome measures for assessing symptoms and functioning in patients with heart failure

**DOI:** 10.1007/s11136-020-02537-y

**Published:** 2020-05-28

**Authors:** Olga Moshkovich, Katy Benjamin, Katie Hall, Ryan Murphy, Robyn von Maltzahn, Boris Gorsh, Vanja Sikirica, Rajnish Saini, Dennis Sprecher

**Affiliations:** 1 ICON Patient Centered Outcomes, 820 W Diamond Ave, Suite 100, Gaithersburg, MD 20878 USA; 2 GSK, Value Evidence and Outcomes, Stockley Park, Uxbridge, Middlesex UK; 3 GSK, Value Evidence and Outcomes, Collegeville, PA USA; 4 GSK, R&D Future Pipeline Discovery Unit, Collegeville, PA USA; 5 GSK, R&D Metabolic Pathways and Cardiovascular Unit, Collegeville, PA USA; 6 Present Address: AbbVie, Chicago, IL USA; 7 Present Address: University of Edinburgh, Edinburgh, UK; 8 Present Address: PHI Inflammation & Immunology, Pfizer Inc., Collegeville, PA USA; 9 Present Address: BioView Consultants LLC., Blue Bell, PA USA

**Keywords:** Heart failure, Patient-reported outcome, Conceptual model, Questionnaire development, Content validity, Concept elicitation

## Abstract

**Purpose:**

Heart failure (HF) is a common condition that places considerable burden on patients. We aimed to develop a patient-reported outcome (PRO) measure to assess the symptoms and impacts of HF.

**Methods:**

Phase 1: a targeted literature review, expert interviews, and concept elicitation (CE) interviews with patients with HF (*n* = 26) were used to develop a conceptual model of the core symptoms and impacts of HF. To capture these concepts, three new fit-for-purpose PRO questionnaires were constructed in accordance with US Food and Drug Administration PRO guidance. Phase 2: three ‘waves’ of cognitive interviews were conducted with patients with HF (*n* = 28) to validate and refine the questionnaires.

**Results:**

Three key symptoms—shortness of breath, oedema, and fatigue—were identified across the literature review, expert interviews and CE interviews. Several additional symptoms, cognitive changes and impacts of HF were reported in the CE interviews and included in the conceptual model. A 10-item symptom questionnaire (Heart Failure-Daily Symptom Diary) was constructed; cognitive testing showed that the final PRO measure was easy to understand/complete and relevant to patients with HF, confirming content validity. Two HF impact questionnaires were developed (Assessing Dyspnoea’s Impact on Mobility and Sleep and Heart Failure-Functional Status Assessment), but required refinement to ensure patient understanding.

**Conclusions:**

Patient input contributed to the development of a PRO instrument for assessing physical and cognitive symptoms important to patients with HF using novel measurement strategies. Inclusion of daily metrics offers differentiation from other qualified instruments and may provide clinical insight for improving lifestyles. Additionally, two draft PRO measures may, after further validation, be useful to assess the impacts of HF.

**Electronic supplementary material:**

The online version of this article (10.1007/s11136-020-02537-y) contains supplementary material, which is available to authorized users.

## Introduction

Heart failure (HF) presents as a cluster of clinical symptoms caused by structural and functional defects in the myocardium, most commonly the left ventricle, resulting in impaired ventricular filling or ejection [1]. It is a common condition that affected an estimated 2.5% of the population of the United States of America (USA) aged ≥ 20 years between 2011 and 2014 [2]. It imposes a high lifetime cost [3], with considerable burden on the patient and healthcare system. The prevalence of HF in the USA is predicted to increase, reaching an estimated 8.5 million people by 2030 [4]. HF often impairs patients’ health-related quality of life (HRQoL) as the symptoms can impact on their ability to carry out daily activities [5, 6] and leads many to enter the hospital for treatment [3].

Most patients with HF experience acute episodes, known as acute decompensated HF (ADHF), which present as a sudden worsening of symptoms [7]. ADHF can indicate deterioration of heart function and often requires urgent medical attention, representing one of the main causes of hospitalisation in patients with HF [7, 8]. Given appropriate medical management, patients can become symptomatically stable following an acute exacerbation [7], although they may continue to experience HF symptoms. It is not clear whether the symptoms and related functional impairments experienced immediately following an ADHF episode and those occurring during stable chronic periods are equivalent.

To quantify patient perceptions of the symptom burden and functional impact of HF, and objectively assess patient status and response to treatment, numerous HF-specific patient-reported outcome (PRO) measures have been published [9]. The US Food and Drug Administration (FDA) provides guidance on the development of new PRO measures, or evaluation of existing measures, to establish suitability to support medical product labelling claims, including ensuring it assesses concepts relevant to patients, and is consistently interpreted as intended and well understood [10]. At the time of this study there was a lack of disease-specific PRO measures for patients with HF that were fit-for-purpose and developed in accordance with the FDA guidance [9, 10]. During writing of this manuscript, two PRO measures (the Kansas City Cardiomyopathy Questionnaire [KCCQ] [11] and the Minnesota Living with Heart Failure Questionnaire [MLHFQ] [12]) have been qualified by the FDA [13, 14], but have limitations as discussed later.

The aim of this study was the identification, adaptation, or construction of a PRO measure to assess both the symptoms of HF and their impacts on patients’ lives, which can eventually be used in clinical studies or clinical practice. Specifically, the objectives were to: (1) develop a conceptual model of the symptoms and impacts experienced by patients with chronic HF following recent hospitalisation for ADHF (acute patients), and in those without a recent ADHF hospitalisation (stable patients); (2) identify, modify, or develop a set of PRO measures to assess the core symptoms and impacts associated with HF in the period immediately prior to and following discharge from hospital for ADHF (acute patients), as well as in stable patients; and (3) perform cognitive testing to ensure instrument content validity. Based on the developed conceptual model and the analysis of existing PRO measures available at the time, we chose to develop new measures that assessed both the symptoms and impacts of HF, including the specific impact of lung congestion (such as shortness of breath [SOB]), and that incorporated a short recall period.

## Methods

### Study design

**Fig. 1:**
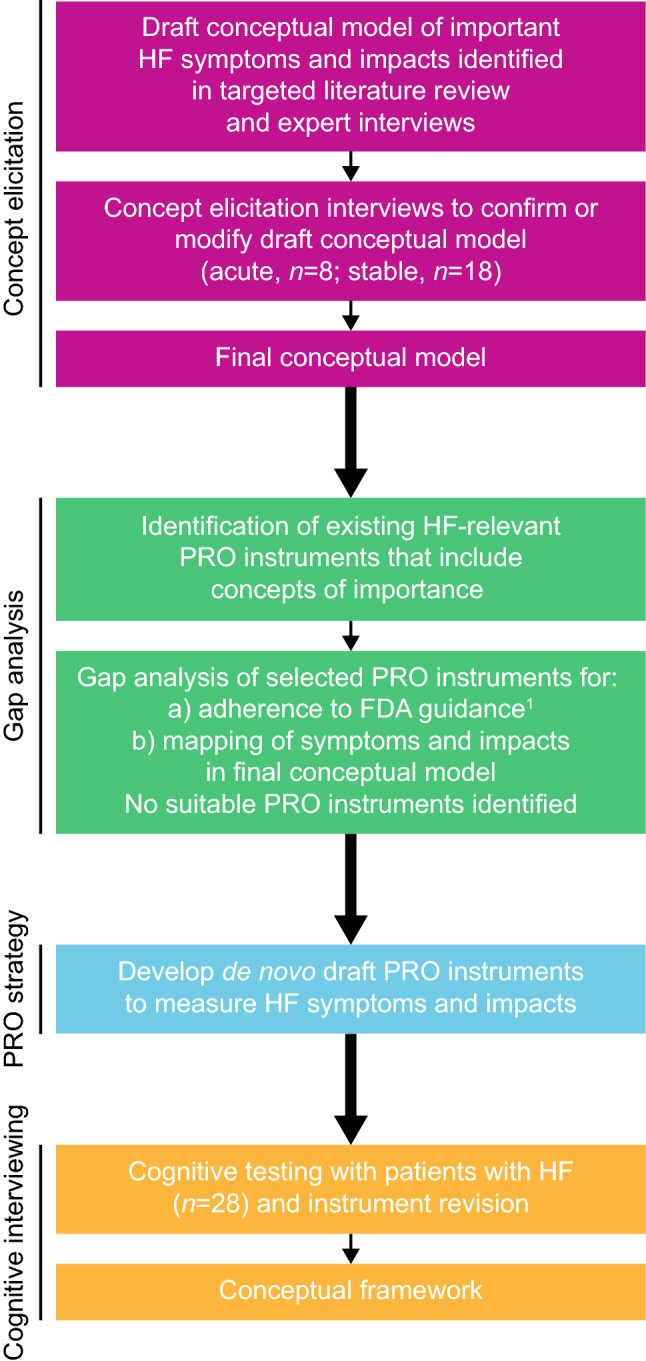
HF PRO instrument development process. 1. US Department of Health and Human Services. Guidance for Industry. Patient-Reported Outcome Measures: Use in Medical Product Development to Support Labelling Claims. 2009; https://www.fda.gov/downloads/drugs/guidances/ucm193282.pdf. Retrieved May 2018. *ADHF* acute decompensated heart failure, *FDA* Food and Drug Administration, *HF* heart failure, *PRO* patient-reported outcome

The study (HO-14-15054/206530) consisted of two main phases. Phase 1 identified the HF symptom and impact concepts important to patients for inclusion in an HF-specific PRO instrument; the findings were used to design PRO measurement strategies and develop a conceptual model. In Phase 2, the draft PRO instruments were tested in patients with HF and refined; based on these results, a conceptual framework was generated. The key steps involved in the development of the conceptual model, PRO measures, and conceptual framework are presented in Fig. 1. The conceptual model (or hypothesised disease model) represents the disease, helping to organise and visualise the various concepts that are relevant to the patient population, and potentially aiding in the selection of endpoints for clinical trials or targets for treatment. In contrast, a conceptual framework is instrument-specific and describes the concepts measured by the PRO instrument in a diagram illustrating relationships between items/concepts [10, 15, 16]. The study’s ethical approval and consent information is presented in the Compliance with Ethical Standards section.

### Phase 1—literature review, expert interviews, and CE interviews

The initial project phase comprised a targeted literature review and expert interviews to develop a draft conceptual model of HF symptoms and impacts. Concept elicitation (CE) interviews involving patients with HF (acute and stable) were conducted to verify that these symptoms and impacts were important and relevant to patients.

Targeted literature searches were conducted in Medline and Embase to identify English-language publications from 2004 to 2014 pertaining to adults with HF-related hospitalisations or ADHF that reported on symptoms, impacts, functioning and/or QoL related to disease burden, or included a PRO measure utilised for patients with HF/ADHF. Case reports, letters and editorials were excluded. A search of the clinicaltrials.gov website was also conducted to identify studies that included HF-related PRO measures. Results were limited to Phase II, III or IV trials of adult patients conducted between 2011 and May 2016. These results were used to inform the expert interview guide and provide insights into potential symptoms and impacts to include in a HF-specific PRO strategy. The literature review, together with the clinicaltrials.gov website search, also identified PRO measures used in HF-related research.

Literature review results and interviews with three leading clinical experts in the diagnosis and treatment of HF (two practising in the USA and one in the UK; Supplementary Materials) identified the HF symptoms and impacts of greatest potential importance to patients; these findings were used to develop a draft conceptual model.

#### CE interview participants

Participants were ≥ 18 years old and recruited from three clinical sites in the USA. Eligibility criteria included the following: diagnosis of chronic HF (≥ 2 healthcare provider visits for HF in the past 2 years), AND discharge from an ADHF-related hospitalisation within 45 days of screening (‘acute’ subgroup) OR no ADHF-related hospitalisations in the past 6 months and no unplanned medical encounter due to HF in the past 3 months (‘stable’ subgroup); left ventricular ejection fraction ≥ 10%; New York Heart Association (NYHA) Functional Class I (up to 20% of the stable patients enrolled), II, III or IV; community dwelling. Exclusion criteria are listed in the Supplementary Materials.

#### CE interviews

The interviews followed a semi-structured guide focusing on the symptoms of chronic HF and ADHF (Supplementary Appendix 1). Patients were asked to report both past and current symptoms. For each symptom reported, the patients were asked about the frequency, duration, severity, level of bother, and impact on their daily lives and functioning. The questions were primarily open-ended but included probes to rate the level of severity and level of bother on a scale of 0–10, where 0 is “not bad/bothersome at all” and 10 is “as bad as it gets/extremely bothersome” to provide additional insights. Interviewers probed specifically for each symptom not mentioned spontaneously by the patient. Interviews were conducted between November 2015 and June 2016. Data were analysed using MaxQDA (VERBI GmbH, Berlin, Germany), a qualitative research software program, and a thematic analysis was conducted to identify concepts of importance to measure in studies of patients with HF. The draft conceptual model was adjusted to reflect CE interview findings and a preliminary PRO questionnaire strategy was developed.

#### Gap analysis

A gap analysis was conducted of HF-related PRO instruments identified in the literature review and search of clinicaltrials.gov (as previously described). The content of the PRO instruments was mapped to the symptoms and impacts included in the final conceptual model to evaluate item coverage and identify gaps in available instruments.

### Phase 2—cognitive interviews and generation of content-valid PRO instrument

Cognitive interviews with patients with HF were conducted to test the PRO questionnaire(s) selected/adapted or developed in Phase 1. The interviews were designed to confirm the content validity of the PRO measure(s) in terms of patient understanding, ease of completion and the relevance of the items/response options to the patients.

Eligibility criteria for the cognitive interviews were similar to the CE interviews, but also included patients with their last ADHF-related hospital discharge 45 days to 6 months prior to enrolment. Interviews were conducted in three waves between December 2016 and November 2017. Patients completed the PRO questionnaires and then took part in a semi-structured cognitive interview, designed to elicit feedback on each PRO overall and obtain item-level feedback. Following each of the first two waves, transcripts were qualitatively analysed; and each PRO item evaluated for clarity, applicability of response options, participant interpretation and relevance. These analyses informed any necessary revisions to the PRO instrument instructions, format, and items between waves, with changes tested in the subsequent wave to achieve a content-valid measure of HF patient-centred outcomes.

## Results

### Literature review

Of 687 articles identified in the literature search, 40 eligible publications were included in the analysis (Supplementary Materials; Supplementary Fig. 1). The three most commonly reported HF and ADHF symptoms were SOB/dyspnoea, fatigue/tiredness/anergia, and oedema/swelling. Other common symptoms included the following: depression, cognitive difficulties, pain, poor appetite or anorexia, nausea/vomiting/diarrhoea, and dizziness/light-headedness. The most frequently reported impacts of HF symptoms were: decreased ability to exercise or perform physical activities, difficulty sleeping or poor sleep quality, and poor overall HRQoL.

### Expert interviews

Clinical experts reported that most of their patients with ADHF were elderly (> 70 years old) and presented with a range of comorbidities, which may have contributed to the complexity of their disease. All three experts identified SOB/dyspnoea, oedema/fluid overload, and fatigue as key symptoms for their patients. SOB and fatigue had the greatest impact on ability to perform daily activities; the impact of oedema was less clear. Additional symptoms mentioned included chest pain, cough, wheezing and weakness.

### CE interviews

Twenty-six patients participated in CE interviews (Table 1), including 18 stable and 8 acute patients. The majority of patients (92%) had ≥ 1 comorbidity, most commonly hypertension, atrial fibrillation, or type II diabetes mellitus.

**Table 1:** Patient demographics, CE, and cognitive interviews

Characteristic	CE interviews (*n* = 26)	Cognitive interviews (*n* = 28)
Gender, *n* (%)		
Male	17 (65)	18 (64)
Female	9 (35)	10 (36)
Age category in years, *n* (%)		
< 50	2 (8)	7 (25)
50–59	4 (15)	11 (39)
60–69	10 (38)	5 (18)
≥ 70	10 (38)	5 (18)
Age range, years	41–90	30–80
Mean age (SD), years	67.8 (12)	56.6 (14)
Race/ethnicity, *n* (%)		
White	16 (61)	8 (29)
Black	8 (31)	18 (64)
Other/mixed	2 (8)	1 (4)
Hispanic/Latino	2 (8)	3 (11)
Main activity, *n* (%)		
Employed (full time or part time)	5 (19)	5 (18)
Retired	14 (54)	8 (29)
Temporary or permanent disability	6 (23)	12 (43)
Other^a^	1 (4)	3 (11)
Marital status^b^, *n* (%)		
Married	17 (65)	10 (36)
Widowed	5 (19)	2 (7)
Divorced	2 (8)	6 (21)
Single, never married	2 (8)	9 (32)
Educational level^c^, *n* (%)		
Less than high school	–	5 (18)
High school diploma or GED	–	8 (29)
Some college/2-year degree	–	11 (39)
Bachelor’s degree or higher	–	3 (11)
Living status, *n* (%)		
Living with partner/spouse	18 (69)	11 (39)
Living alone	3 (12)	8 (29)
Living with other family members	5 (19)	7 (25)
Other^d^		2 (7)

*CE* concept elicitation, *GED* general education development, *SD* standard deviation

^a^Includes options unemployed, student, or unspecified

^b^One participant (Wave 3) in the CE study did not specify marital status

^c^Educational level data were not collected in the CE study and was unspecified for 1 participant of the cognitive interviews

^d^Includes roommates or unspecified

Most patients (acute and stable) reported experiencing dyspnoea (*n* = 25, 96%), fatigue (*n* = 22, 85%) and oedema (*n* = 23, 88%) (Tables 2 and 3). They described their dyspnoea as “*the worst feeling ever*”; swelling like “*dragging cinder blocks*”; and with fatigue, “*your body just wants to give out*” (Tables 2 and 3). Other symptoms were reported by fewer patients (*n* ≤ 6): SOB while lying flat (orthopnoea), chest pain, cough, weakness, wheezing, heart palpitations and dizziness. In addition, cognitive changes, including memory problems and difficulty concentrating, were reported by 14 (54%) patients.

**Table 2:** CE interviews: physiological symptom summary in the acute subgroup (*n* = 8)

Symptom	Sample, *n* (%)	Frequency, *n*	Duration, *n*	Severity, range, (scale 0–10^a^)	Level of bother, range (scale 0–10^b^)	Impacts reported	Patient description
Oedema	8 (100) Spontaneous report: 6 Current symptom^c^: 5	Daily: 2 Weekly: 2 Monthly: 1	Day: 1 Days–week: 1 Until medication taken: 1 Until patient urinates: 1	Hospital admission: 5–10 Discharge: 0–4 Now: 0–10	2.5–8	Walking, diet, self-care activities	“…it was like I was dragging cinder blocks on my legs”.
Dyspnoea	7 (88) Spontaneous report: 5 Current symptom^c^: 3	Daily: 3 Activity dependent: 1	1–15 min	Hospital admission: 6–10 Discharge: 0–7 Now: 0–6	3–8	Walking/climbing stairs, sleep, ADL, recreation, fitness, emotional, social	“…that is the worst feeling ever, the shortness of breath, because you’ve got to gasp, you’ve got to look for air…it’s almost like you’re out of shape. Once you do it and it passes, you feel real good, you feel like you just ran a marathon or something…until the next time”.
Fatigue	6 (75) Spontaneous report: 4 Current symptom^c^: 5	Daily: 5	30 min–constant	Hospital admission: 7–9 Discharge: 1 Now: 1–9	5–10	Walking, social, recreation, ADL	“I walked from my car, leaning on the building, leaning on cars, I’m like what the hell is wrong with me? This is not me at all. Leaning on the side of the building, people would catch me”.
Chest pain	3 (38) Spontaneous report: 3 Current symptom^c^: 3	Daily: 2 Varies: 1	–	Hospital admission: 5 Discharge: 5 Now: 3–10	–	ADL, sleep, walking	“It’s in the area where my heart is… what was most uncomfortable was the piercing in the back…”
Cough	2 (25) Spontaneous report: 2 Current symptom^c^: 0	In hospital only: 2	2 min–constant	Hospital admission: 8 Discharge: 5 Now: 1–2	–	Recreation, walking	“I had a cough, a constant cough and it was very annoying”.
Weakness	1(13) Spontaneous report: 1 Current symptom^c^: 1	–	–	–	–	ADL, walking	“I was terribly weak. Very, very weak and it frightened me because I wanted to be able to get up and walk around, but I could only walk just a few steps and I was done”.

ADL, activities of daily living

^a^0 is “not bad at all” and 10 is “as bad as it gets”.

^b^0 is “not at all bothersome” to 10 is “extremely bothersome”.

^c^Patients reported current and past symptoms. Current symptoms are those a patient is currently experiencing. Spontaneous symptoms are those that were mentioned without probing from the interviewer

Only symptoms/impacts reported by at least 3 patients in the entire sample are included. Not all patients who reported a symptom provided information on each dimension (e.g. frequency and duration)

Saturation was not reached in the acute patient subgroup, as several patients reported novel symptom concepts through the 8th interview; however, no additional interviews with acute patients could be completed due to recruitment challenges

**Table 3:** CE interviews: physiological symptom summary in the stable subgroup (*n* = 18)

Symptom	Sample, *n* (%)	Frequency, *n*	Duration, *n*	Severity, range, (scale 0–10^a^)	Level of bother, range, (scale 0–10^b^)	Impacts reported	Patient description
Oedema	15 (83) Spontaneous report: 4 Current symptom^c^: 9	Daily: 4 Weekly: 1 Monthly: 1 Rarely: 5	Constant: 1 Day/couple of days: 2 Until legs elevated: 2 Oedema dependent:2	Now: 1–10	2–10	Walking, diet, self-care activities	“…if I sit for a long period of time, my ankles will look like grapefruits. My toes will look like… Flintstone toes, and so they just real fat and pudgy…” “…it’s like walking on clouds… it’s no traction with it”.
Dyspnoea	18 (100) Spontaneous report: 16 Current symptom^c^: 15	Daily: 8 Weekly: 3 Activity dependent: 2 Other: 4	< 1 min: 5 2–5 min: 6 Up to 1 h: 1 Other: 2	Now: 0–8	0–10	Walking/climbing stairs, sleep, ADL, recreation, fitness, emotional, social	“It starts like somebody is sitting in your chest and stomach…a fish out of water and you’re trying to breathe any gulp of air…You just cannot get enough oxygen in your system”.
Fatigue	16 (89) Spontaneous report: 11 Current symptom^c^: 14	Daily: 11 Activity dependent: 1 Other: 2	Constant: 4 < 10 min: 2 Hours–day: 2 Alleviates after resting: 3 Varies: 1	Now: 1–8	5–10	Walking, social, recreation, ADL	“Your body just wants to give out. You don’t want to do anything…You know you have to stop and either sit down or lay down and it will pass, so that’s what I do”.
Chest pain	2 (11) Spontaneous report: 1 Current symptom^c^: 2	On exertion: 1 Medication dependent: 1	< 10 min: 2	Now: 2–10	0–10	–	“Well, I can remember the first heart attack I had, which was in a hospital following a cardiac procedure, and…it went right to a 10”.
Cough	1 (6) Spontaneous report: 1 Current symptom^c^: 1	Daily: 1	A few seconds	Now: 2–4	2^d^	Psychological	“At its mildest it’s just a couple of little hacks, kind of like you’ve got a frog in your throat and at its worst you cough three or four times and cough up a pretty good size amount of fluid out of your lungs”.
Weakness	5 (28) Spontaneous report:5 Current symptom^c^: 2	Daily: 2	Constant: 1	–	10^d^	Walking, lifting items, employment, psychological	“Ah, lack of stamina, lack of strength”.
Heart palpitations	4 (22)	Once: 1 Weekly: 1 Activity dependent: 2	1 h or longer: 2 A few minutes: 1 A few seconds: 1	Now: 7–10	7–10	Psychological, ADL, leaving the house	“It beats real fast and it skips beats…at my worst you literally…see that my heart is beating real fast, it’s palpitating real fast”. “…when it pauses… it takes my breath”.
Light headedness/dizziness	4 (22)	Once: 1 1–2 × weekly: 1 ≥ 3 × weekly:1 Prior to last hospitalisation: 1	20 min: 1 Not stated: 3	–	8^d^	Psychological, sleep, driving, walking	“It is sometimes it feel like the room is spinning and I don’t know if I have to puke or not”.

*ADL* activities of daily living

^a^0 is “not bad at all” and 10 is “as bad as it gets”.

^b^0 is “not at all bothersome” to 10 is “extremely bothersome”.

^c^Patients reported current and past symptoms. Current symptoms are those a patient is currently experiencing. Spontaneous symptoms are those that were mentioned without probing from the interviewer

^d^Reported by one patient

Only symptoms/impacts reported by at least 3 patients in the entire sample are included

Saturation was reached in the stable patient subgroup by the 12th of 18 interviews

Patients reported impacts of their HF symptoms on physical functioning, including difficulties in performing daily functions (e.g. walking, climbing stairs, sleeping) and activities (e.g. showering, household chores, sports/hobbies). Most patients (*n* = 23, 88%) also reported at least one emotional impact. Such impacts were directly related to specific symptoms or due to limitations caused by HF, and included frustration, worry about the future, depression, irritability, fear, and anxiety.

Few differences in the core symptoms were identified between stable and acute patients, either in terms of the HF symptoms experienced at the time of their interview or at hospitalisation. Acute patients generally had higher severity ratings versus stable patients, and a narrower range of severity for dyspnoea and oedema (Tables 2 and 3).

Nearly all symptom concepts were reported by the interview of the 18th patient, except for one new symptom reported by the final patient interviewed (numbness in hands), which is not typically associated with HF. These findings indicate that concept saturation was reached in terms of condition-specific symptoms. Saturation was also assessed by subgroup; all symptom concepts were reported by the interview of the 12th patient in the stable subgroup, but saturation was not reached when considering the acute patients only.

### Final conceptual model

**Fig. 2:**
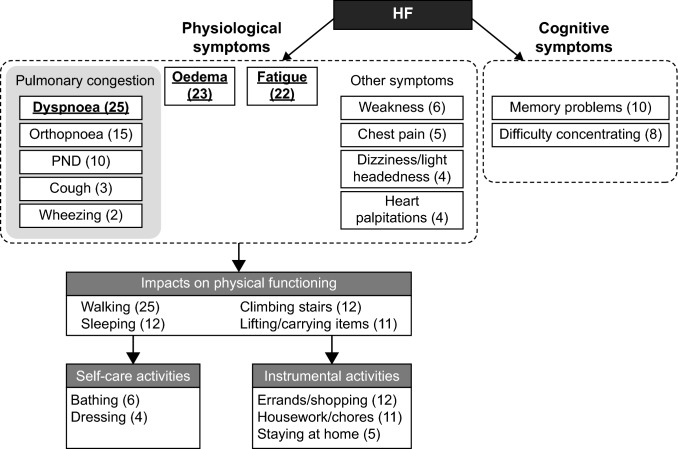
Final conceptual model. The numbers in parentheses denote the number of patients who reported the symptom/impact in the sample. Underlined symptoms were the most prevalent. *HF* heart failure, *PND* paroxysmal nocturnal dyspnoea

Based on the results from the literature review, expert interviews, and CE interviews, a final conceptual model was developed. The final conceptual model identifying the key concepts important to patients with HF is shown in Fig. 2. The model focused on symptoms, as well as proximal impacts (i.e. those expected to change as symptoms improve or worsen and that are directly related to HF). More distal impacts (e.g. financial burden or emotional impacts) were excluded. One model was developed for both acute and stable patients, due to the similarity in concepts identified.

### Gap analysis and PRO strategy: rationale for a novel PRO measure

Nine HF-related PRO measures were identified in the literature review and the clinicaltrials.gov website search; the results of the gap analysis for all instruments are shown in Supplementary Table 1. The most commonly used PRO instruments identified in both the literature review and the clinicaltrials.gov search were the MLHFQ [17, 18] and the KCCQ [11]. The gap analysis (Supplementary Table 1) revealed that neither instrument captured all important concepts identified in the final conceptual model. Both measures assess the three sentinel symptoms of heart failure—dyspnoea, oedema, and fatigue—but do not assess weakness, chest pain, dizziness, palpitations, nausea, weakness, or cough. The MLHFQ, which is the most widely used in this population, also includes items on employment status, hospital stay, and financial impact; while these are confirmed impacts of HF in the bigger picture of HRQoL, they are unlikely to be sensitive to treatment effect within a clinical trial setting. Additionally, neither of these instruments measure symptom severity or intensity directly, instead asking about degree of symptom bother (KCCQ) or degree of limitation caused by the symptoms (MLHFQ). Furthermore, both the KCCQ and MLHFQ utilise long recall periods (2 weeks and 4 weeks respectively) for the assessment of symptoms. Finally, a previous review confirmed that neither measure satisfied all the recommendations for evidence of validity as outlined in the 2009 FDA PRO guidance for labelling claims [9]. In addition, while the MSAS-HF has items within most of the relevant symptoms, it does not include mobility-related impacts or impacts on daily activities, and includes items on emotional burden and physiological symptoms that are not part of the conceptual model. As considerable revision would be required to adapt an existing instrument, it was decided to proceed with de novo questionnaire development.

Three new PRO instruments were developed. The first (the Heart Failure-Daily Symptom Diary [HF-DSD]) was developed to measure the frequency, duration and severity of 10 HF symptoms (SOB/dyspnoea, orthopnoea, chest pain, coughing, wheezing, oedema, tiredness, weakness, dizziness, and palpitations). As the CE interviews showed that symptoms can change on a daily basis, even among stable patients, the symptom measure was designed for daily administration (past 24 h). In contrast, most of the existing measures include a recall period of ≥ 1 week (Supplementary Table 1).

The other two new instruments were developed to assess the symptom impacts identified in the conceptual model and were designed for weekly administration (past 7 days). Given that SOB was reported to be one of the most impactful symptoms, one instrument, the Assessing Dyspnoea’s Impact on Mobility and Sleep (ADIMS) questionnaire focussed on the effects of this symptom on daily life. Another instrument, the Heart Failure-Functional Status Assessment (HF-FSA), consisted of two domains assessing (1) the impact of HF symptoms on day-to-day activities, for example, getting dressed or doing housework; and (2) changes in cognitive abilities. Further details of the questionnaires are provided in the Supplementary Materials.

### Cognitive interviews

Twenty-eight patients with HF participated in the cognitive interviews (Table 1), with 11, 8, and 9 participants in Waves 1, 2, and 3, respectively. The majority were classified as NYHA Class II (57.1%) or III (35.7%), but the sample also included one Class I and one Class IV patient. The most common comorbidities were hypertension (75.5%), diabetes (46.4%), and arthritis (17.8%). Across all waves, about a third (28.6%) of patients had been discharged from their last ADHF hospitalisation within 45 days of the interview and an equal percentage had been discharged within the past 6 months; the remainder had stable HF.

#### HF-DSD

All Wave 1 patients (*n* = 11) found the HF-DSD easy to complete overall. The majority of patients found the 0–10 NRS scale appropriate and interpreted the anchors as expected. Although most patients found each item easy to understand, patients did not clearly differentiate between different dimensions of a symptom (i.e. frequency vs duration vs severity). Consequently, in preparation for Wave 2, the PRO was simplified to assess only the severity of each symptom, “how bad… at its worst”, resulting in a 10-item questionnaire. When asked about the recall period, the majority (*n* = 9) of patients reported thinking of the past 24 h (the remaining 2 participants considered longer amounts of time).

All patients in Wave 2 (*n* = 8) found the questionnaire easy to complete, and only minimal changes to item wording were made between Waves 2 and 3.

Overall, patients in Wave 3 (*n* = 9) found the questionnaire to be a good instrument to evaluate the symptoms of HF (“…I could see myself in the questions”). All patients reported the instrument was clear and easy to complete. All patients reported willingness to complete the questionnaire daily for 1 month, and 6 patients reported that completing it for 6 months would be acceptable. No additional changes were recommended based on results from Wave 3. Across all waves, most symptom concepts were reported to be relevant by a majority of the sample, and no additional concepts (reported by > 2 patients) were identified for potential inclusion. A summary of item revisions across the three waves is shown in Supplementary Table 2.

#### ADIMS questionnaire

Most patients across all waves reported that the ADIMS questionnaire was relevant, easy to understand and complete, and no new concepts were added based on patient feedback. Of the 11 patients in Wave 1, only 5 reported using the correct recall period of the past week. Therefore, the term “over the past 7 days” was added to each root question in bold. In Wave 2, most (*n* = 7/8) patients reported a recall period in line with the past week; the other patient did not notice the recall instruction and so an introductory sentence was added to emphasise the recall period: “The next questions ask about your shortness of breath while doing various activities in the PAST 7 DAYS”. In Wave 3, 7/9 participants used the correct recall period.

Minor changes to the wording of the sleep impact items, and an updated response scale for frequency of sleep difficulties, were implemented between waves; results from Wave 3 indicated that no further changes were required in this domain. When completing the mobility items, participants could not differentiate between two response options: inability to do the activity because of SOB and not having the opportunity to do the activity in the past 7 days, informing changes to the item wording and response scales between waves. These concerns were not fully resolved with revisions at Wave 3; thus, the item structure and response scale was further modified by using a two-part structure: a dichotomous (yes/no) gating item asking if the patient had performed the activity in the past 7 days and, for those who had, a second item rating the impact of the HF symptom on their performance. Furthermore, approximately one-third of patients across all waves thought of having to stop and take a break from the activity in their definition of options between “mild” and “severely”, in contradiction to the “…without stopping” wording originally included in each item. Therefore, the final versions of the item response options did not specify “without stopping”. A summary of item revisions across the three waves is shown in Supplementary Table 3.

#### HF-FSA

Cognitive testing of HF-FSA confirmed that the included daily activities concepts were relevant to most patients with HF. One concept (housework requiring heavy effort) was added to the measure after the first wave. Furthermore, the wording was revised based on Wave 1 feedback to ensure consistency across all activity items, and with the items in the ADIMS questionnaire (e.g. including the recall period in the root question of each item). As with the ADIMS cognitive testing, Wave 3 results indicated that some patients were unable to distinguish between “unable to do” and “I did not do this in the past 7 days”; therefore, the activity concepts were separated into a gating question (was the activity done in the past 7 days) and a rating question (difficulty level for the activity), consistent with final changes to the ADIMS questionnaire.

Across all waves, the cognitive ability items “ability to concentrate” and “ability to remember things” were reported as relevant by 8/16 (50%) and 6/15 (40%) participants, respectively. The cognitive ability items were retained across all 3 waves and generally found to be easy to understand and were interpreted appropriately. In the final wave, several patients indicated confusion/misinterpretation around the most extreme response option “not at all”; therefore, this option was removed. No other changes to the cognitive ability domain were required following the third wave. A summary of item revisions across the three waves is shown in Supplementary Table 4.

#### Conceptual framework

**Fig. 3:**
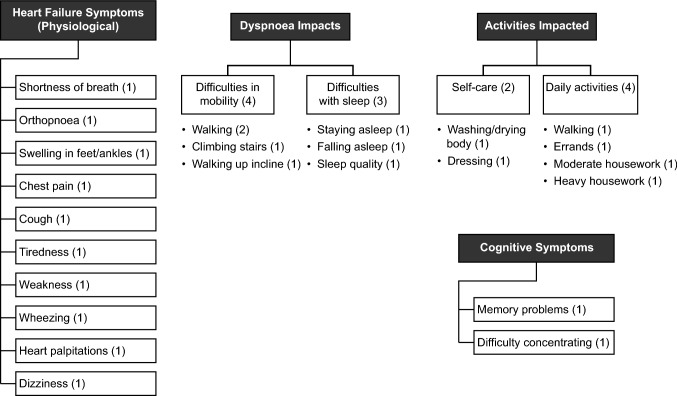
Conceptual framework. The numbers in parentheses denote the number of items under each domain

The conceptual framework for the three PRO measures to evaluate the symptoms and impacts of HF is presented in Fig. 3. This was developed, based on the final conceptual model and the cognitive interview outcomes, to depict the overall construct of HF-related symptom burden, the domains representing this construct and the items that measure each domain.

## Discussion

ADHF and chronic stable HF manifest as a variety of symptoms and affect multiple areas of patients’ physical functioning and ability to perform activities of daily living, as illustrated in our conceptual model. At the time this study was conducted (prior to 2016), there was an absence of a fit-for-purpose PRO instrument to measure patient experience of HF that met FDA guidance [9, 10]. Therefore, we developed a daily 10-item PRO instrument in accordance with FDA guidance to measure the key symptoms associated with HF, utilising a short, 24-h recall period and draft self-report instruments to measure the impacts of HF.

An important outcome of this study was the development of a conceptual model for HF that considers both symptoms and impacts. The development of the conceptual model and the gap analysis was based upon three types of evidence: review of published peer-reviewed literature, in-depth interviews with three clinical experts and CE interviews with 26 patients with HF. A consistent picture emerged of the important symptoms associated with HF and ADHF across all three sources, indicating a high content validity of the PRO measurement strategy. The three core symptoms identified—SOB/dyspnoea, fatigue and oedema—caused considerable impairment in patients’ physical functioning, affecting all areas of their lives. While SOB was identified as a key symptom leading to functional impairment, it should be noted that these impacts may be exacerbated by other symptoms, such as fatigue. Few differences in the core symptoms were found between patients with acute and stable HF, suggesting that the types of symptoms do not vary substantially by disease status, but are more marked in patients with more severe disease. More subtle differences may not have been detected due to the small sample size of the acute subgroup in our CE study; clinical differences could be further explored in a larger study.

Due to the complex pathophysiology of HF, the range of symptoms can be difficult to capture as early stages of HF are asymptomatic or lack specific signs [1]. The CE interviews showed that the intensity of HF symptoms can also vary day-to-day, highlighting the complex nature of the disease and the need to measure multiple concepts to holistically assess HF exacerbations and their treatment efficacy. This daily heterogeneity is large enough to suggest that a valuable endpoint in clinical studies would be an increase in the number of “better” days provided by a therapeutic approach. PRO measures with a longer recall period may exclude key patient-relevant information by averaging the patient experience over a number of weeks.

In addition to physiological symptoms, the conceptual model included physical limitations related to symptom burden, and the tasks and activities most proximally affected by these symptoms. The primary impacts of HF symptoms were difficulty walking, climbing stairs, sleeping and lifting/carrying objects; each of these activities places stress on the body by causing changes in internal pressures (posture/sleeping) or cardiac output (work). All patients participating in the CE interviews reported difficulty walking as an impact of their disease, while only half reported difficulty climbing stairs. This discrepancy should be investigated in larger studies to determine if some patients do not report difficulties in climbing stairs due to HF-related lifestyle adaptations (e.g. by avoiding stair climbing situations or environments).

In the cognitive interviews, while only 50% and 40% of patients, respectively, reported problems with “ability to concentrate” and “ability to remember things” as relevant, this was consistent with the proportion of patients in the CE interviews who self-reported cognitive issues and therefore the concepts were retained. It is recognised that cognitive issues may be experienced by many elderly individuals, making them difficult to equate directly to HF; future studies may compare these results to those of the questionnaire completed by age-matched healthy volunteers.

More distal impacts, such as financial burden or emotional problems, were excluded from the conceptual model. While we acknowledge the importance of these impacts on overall QoL, such outcomes generally have multi-faceted causation and have no definitive, direct association with HF [12, 19]. However, these concepts may be relevant for the development of alternative PRO tools.

The patient perspective is increasingly viewed as important when evaluating treatment efficacy and making treatment decisions. Valid, fit-for-purpose PRO instruments are needed to enable physicians and researchers to understand the symptoms and impacts of HF most relevant to patients in order to improve their health and HRQoL. Our review of the literature did not identify any existing HF-specific PRO instruments that included all the key concepts of importance to this patient population and that were developed using FDA-recommended methods. Since the conclusion of this study, the two most commonly used questionnaires (MLHFQ and KCCQ) have been qualified by the FDA [20, 21]. While they are valuable additions to the quantification of the patient experience, these instruments require greater than 24-h recalls and they do not include all concepts identified as important to measure in this population, according to our conceptual model. While both instruments measured the key symptoms of oedema, SOB/dyspnoea and fatigue, neither instrument measured chest pain, cough or lifting/carrying items, and the KCCQ did not include any items addressing cognitive functioning. Furthermore, neither the MLHFQ nor the KCCQ contained a direct measure of symptom severity, rather assessing symptoms by degree of activity limitation (MLHFQ) or level of bother and frequency (KCCQ). Therefore, a new PRO tool to measure HF symptoms (the HF-DSD) was developed, which included new additional concepts and a novel measurement approach. Cognitive interviewing found the final instrument was content-valid for assessment of HF symptoms, and easy to understand and complete. Consistent with FDA guidance, which states that shorter recall periods are preferred [10], this instrument also has the advantage over most other HF-related PRO symptom measures of utilising a 24-h recall, allowing symptoms to be assessed more accurately, and capturing day-to-day fluctuations in severity. This is consistent with the CE data that showed daily variation in HF symptoms. Psychometric analyses will be needed to confirm the reliability and statistical validity of the instrument for the HF context of use (according to FDA guidance [10]). It is also recommended that the instrument be tested in a pilot study or clinical trial to understand how the instrument performs on a daily basis. Future transitions from a paper version to an electronic tool will require equivalence and usability testing.

While the cognitive interviews confirmed the relevance of the two impact PRO instruments to the target population, both required changes to item response options following the third wave. Additional cognitive interviews will be required to assess content validity of these revised versions and their psychometric validity evaluated. Once fully validated, these PRO measures are expected to be valuable additions to the field of HF outcomes research, providing useful information about the consequences of HF and the outcomes of treatment from the patient’s perspective.

Since the development of our PRO instrument, a new measure, the PROMIS-Plus-HF (Patient-Reported Outcomes Measurement Information System®-Plus-Heart Failure) profile measure, has become available [22]. While this tool allows extensive HF evaluation, it is lengthy and its physical symptom questions focus on the impact of SOB and fatigue on various activities, with only one question on swelling (it’s frequency in the past 7 days). PROMIS-Plus-HF has 86 items and is estimated to take 15 min to complete the full instrument; a shorter measure may be preferred to limit the patient burden during clinical trials. Indeed, the authors indicate that work is required to create a short-form version and to develop summary scores for physical, mental and social health aspects. We have proposed PRO measures with short standalone elements, creating low patient burden and allowing independent assessment of symptoms.

A strength of this study was that the patient samples used in the CE and cognitive interviews represented a diversity of races, sexes, ages and disease severities. While there were no patients 20–40 years of age included in the CE interviews, chronic HF generally affects the elderly (as reported in the expert interviews) and HF prevalence increases with age [23]. Therefore, our sample is representative of the HF patient population. In addition, a wide range of ages and educational levels were represented in the patient sample used for cognitive interviews. Therefore, the content validity of the instrument was confirmed in patients with a wide range of characteristics. Each wave of cognitive interviews included patients who were recently discharged following an ADHF episode, and those who have not had a recent ADHF episode, to understand the range of symptom experiences in this population.

Like most qualitative research, the sample size (*n* = 54 across both phases), while adequate to establish content validity, was relatively small; thus, some differences in patient experiences may not have been captured. In addition, the study eligibility criteria may have led to an under-representation of patients at both ends of the severity scale (NYHA Class I and IV). Additional cognitive interview studies with a larger population of patients at these extreme ends of the severity spectrum may be needed to confirm the instruments’ validity in these groups. Furthermore, comorbidities are common in patients with HF [24] and it is challenging to differentiate between HF-related symptoms and those of common comorbidities, such as sleep apnoea [25, 26]. In the present study, most patients had at least one comorbidity; it is possible that some symptoms due to comorbid conditions may have been attributed to HF.

## Conclusions

This study reports the development of a conceptual model and conceptual framework for assessing both HF symptoms and impacts. A fit-for-purpose 10-item PRO questionnaire was constructed that follows FDA guidance [10], has a short (24 h) recall, and is clearly understood by patients with HF. Cognitive testing showed that the HF-DSD is content-valid and appropriate for assessing symptom burden in both patients with acute and stable HF. With confirmation of psychometric properties, this instrument may be used in clinical studies to measure treatment efficacy in a way that is clinically meaningful, as it includes all three elements (dyspnoea, fatigue, and oedema) that are of critical concern to this patient population and permits analyses of daily influence, thereby ultimately improving treatment options/management of patients with HF. It could also be useful in assessing changes in patients’ HF-related health status that may drive treatment decisions. The further development of two HF-specific impact PRO instruments is also in progress. In addition touse in clinical trials, the impact PRO measures with weekly recall could potentially be used in the clinical setting to assess recovery after an episode of ADHF.

## Electronic supplementary material

Below is the link to the electronic supplementary material.

Supplementary file1 (DOCX 253 kb)
