# Correction to: Development of a conceptual model and patient-reported outcome measures for assessing symptoms and functioning in patients with heart failure

**DOI:** 10.1007/s11136-020-02631-1

**Published:** 2020-09-17

**Authors:** Olga Moshkovich, Katy Benjamin, Katie Hall, Ryan Murphy, Robyn von Maltzahn, Boris Gorsh, Vanja Sikirica, Rajnish Saini, Dennis Sprecher

**Affiliations:** 1 ICON Patient Centered Outcomes, 820 W Diamond Ave, Suite 100, Gaithersburg, MD 20878 USA; 2 GSK, Value Evidence and Outcomes, Stockley Park, Uxbridge, Middlesex UK; 3 GSK, Value Evidence and Outcomes, Collegeville, PA USA; 4 GSK, R&D Future Pipeline Discovery Unit, Collegeville, PA USA; 5 GSK, R&D Metabolic Pathways and Cardiovascular Unit, Collegeville, PA USA; 6 Present Address: AbbVie, Chicago, IL USA; 7 Present Address: University of Edinburgh, Edinburgh, UK; 8 Present Address: PHI Inflammation & Immunology, Pfizer Inc., Collegeville, PA USA; 9 Present Address: BioView Consultants LLC., Blue Bell, PA USA

## Correction to: Quality of Life Research 10.1007/s11136-020-02537-y

The article “Development of a conceptual model and patient-reported outcome measures for assessing symptoms and functioning in patients with heart failure”, written by Olga Moshkovich, Katy Benjamin, Katie Hall, Ryan Murphy, Robyn von Maltzahn, Boris Gorsh, Vanja Sikirica, Rajnish Saini and Dennis Sprecher was originally published electronically on the publisher’s internet portal on 28 May 2020 without open access. With the author(s)’ decision to opt for Open Choice the copyright of the article changed on 15th September to © The Author(s) 2020 and the article is forthwith distributed under a Creative Commons Attribution 4.0 International License (https://creativecommons.org/licenses/by/4.0/), which permits use, sharing, adaptation, distribution and reproduction in any medium or format, as long as you give appropriate credit to the original author(s) and the source, provide a link to the Creative Commons licence, and indicate if changes were made.

The original article has been corrected.

