# Supplementary material for: Development of a conceptual model and patient-reported outcome measures for assessing symptoms and functioning in patients with heart failure
Source: Qual Life Res. 2020 May 28;29(10):2835–48. doi: 10.1007/s11136-020-02537-y (PMC7561560; doi:10.1007/s11136-020-02537-y)
Supplement: Supplementary file 1 — Supplementary file1 (DOCX 253 kb) [file 11136_2020_2537_MOESM1_ESM.docx]

**Development of a conceptual model and patient-reported outcome measures for assessing symptoms and functioning in patients with heart failure**

**Olga Moshkovich,^1^ Katy Benjamin,^1*^ Katie Hall,^1**^ Ryan Murphy,^1^ Robyn von Maltzahn,^2^
Boris Gorsh,^3^ Vanja Sikirica,^3***^ Rajnish Saini,^4^ Dennis Sprecher^5****^**

^1^ICON Patient Centered Outcomes, Gaithersburg, MD, USA; ^2^GSK, Value Evidence and Outcomes, Stockley Park, Uxbridge, Middlesex, UK; ^3^GSK, Value Evidence and Outcomes, Collegeville, PA, USA; ^4^GSK, R&D Future Pipeline Discovery Unit, Collegeville, PA, USA; ^5^GSK, R&D Metabolic Pathways and Cardiovascular Unit, Collegeville, PA, USA

*Current affiliation: AbbVie, Chicago, IL, USA

**Current affiliation: University of Edinburgh, Edinburgh, UK

***Current affiliation: PHI Inflammation & Immunology, Pfizer Inc., Collegeville, PA, USA

****Current affiliation: BioView Consultants LLC., Blue Bell, PA, USA

**Corresponding Author:** Olga Moshkovich

**Address:** 820 W Diamond Ave, Suite 100, Gaithersburg, MD 20878, USA

**Tel:** (+1) 301-944-6785

**Email:** Olga.Moshkovich@iconplc.com

**Supplementary Materials**

# Methods

# Literature review

Searches were conducted in Medline and Embase using the search terms shown in the Table below.

**Table. Summary of search strategy terms**

| Search strategy | Search terms |
| --- | --- |
| Databases | - MedlineR In-Process & Other Non-Indexed Citations - Embase |
| Disease terms | - “Heart Failure” (in title)   AND   - [advanced OR decompensated OR worsen] - [hospital OR admitted] - [hospitalization] |
| Outcomes-related terms Group I  (AND disease search terms OR “heart failure” in title) | - Quality of life - HQL or HRQL or HRQoL - Quality of life index - QWB - Quality of well$being AND - Questionnaire$ - Interview$ |
| Outcomes-related terms  Group II | - Sign$ and symptom$ - Symptom$ (title only) - Symptom Assessment - Symptom score$ - Clinical characteristic$ (title only) - Clinical feature$ (title only) - Patient$ characteristic$ (title only) - Scale - Measure |
| Article types  (AND group II outcomes-related terms AND disease terms) | - Epidemiologic studies/ case-control studies/ cohort studies/ cross-sectional studies/clinical trials - Longitudinal or observational studies - Registries - Health surveys - Data collection |
| Specific PRO search terms  (AND “heart failure” in title) | - Heart failure score - Patient reported outcome |
| Search Limits | - Humans - English language - Published 2004 – Current - Not pediatric studies (title only) - Not letters - Not case reports |

Following removal of duplicates, search results were reviewed for the following inclusion criteria: abstract available; adults (≥18 years of age); included heart failure (HF)-related symptoms, impacts, quality of life, disease burden, or a HF-specific patient reported outcomes (PRO) instrument; research articles, reviews, practice guidelines, consensus papers, epidemiologic studies and clinical trial reports. Exclusion criteria were: animal studies; reports on biomarkers/pathology/histopathology; case studies; letters to the editor. Resulting abstracts were reviewed and those retained that included, or were likely to include, information on symptoms, impacts, functional status, quality of life, or descriptions of studies that include PRO, clinician reported outcomes, or observer reported outcomes instruments relevant to the HF patient population.

# Expert interviews

One-to-one telephone interviews were conducted with three clinical experts. Interviews were semi-structured, following an interview guide with open-ended questions. Two of the interviewees were physicians, and one was an advanced practice nurse. Each expert had been practicing medicine for ≥19 years at the time of the interview and had extensive experience in treating patients with acute decompensated HF (15–30 years).

# Concept elicitation (CE) and cognitive interviews: participants

Exclusion criteria for both the CE interviews and cognitive interviews were: a diagnosis of asthma or chronic obstructive pulmonary disease; history of stroke in the past 6 months; history of myocardial infarction within the past 3 months; any major medical event within the past 6 months that could, in the investigator’s opinion, impact the patient’s mobility or activities of daily living (e.g. hip fracture, heart transplant, angioplasty, etc.); a medical or psychiatric illness, or indication of cognitive impairment, that could, in the investigator’s opinion, potentially interfere with the patient’s ability to participate in this interview study. In both the CE and cognitive interviews, patients provided written informed consent prior to being scheduled for an interview and were compensated $125 for each interview.

# Results

**Literature review**

A total of 687 articles were identified from the literature search, of which 16 were missing abstracts; 671 abstracts were reviewed for content and data were extracted from 40 eligible publications and conference abstracts, including four studies identified as relevant by the study sponsor (Supplementary Figure 1). The selected articles included reports of 7 randomised controlled trials, 13 longitudinal studies, 7 qualitative interview studies, 11 cross-sectional studies, one study with a pre-test-treatment-post-test design, and one systematic review [1-40].

**PRO instruments**

***The Heart Failure-Daily Symptom Diary (HF-DSD)***

The instrument was developed to measure ten key symptoms experienced by patients with heart failure (HF). Patients were asked to rate each symptom within a 24-hour recall period using a 0–10 numeric pain rating scale. The initial version developed was composed of 25 items, with multiple dimensions (frequency, severity, and duration) assessed for each symptom. For frequency, 0 = never had the symptom and 10 = symptom always present; for severity, 0 = mild and 10 = very severe; for duration, 0 = lasted only for a moment and 10 = lasted for a very long time. However, based on patient feedback from the first round of cognitive interviews, the PRO instrument was simplified to assess only the severity of each symptom, “how bad… at its worst.”

***The Assessing Dyspnoea’s Impact on Mobility and Sleep (ADIMS) questionnaire***

The instrument was developed to measure key impacts associated with shortness of breath (SOB): mobility (four items related to the degree of SOB experienced during the activity) and sleep impacts (three items related to the frequency of the sleep problems due to HF symptoms). Items were measured using verbal response scales based upon a 1-week recall period. Mobility items were rated on a scale of “no shortness of breath” to “severely short of breath” and included the options “I was unable to do this because of shortness of breath” and “I did not do this in the past 7 days”; items related to sleep difficulty due to SOB were rated on a scale of “never” to “always”.

***The*** ***Heart Failure-Functional Status Assessment (HF-FSA)***

This questionnaire consisted of two domains, assessing A) the impact of any HF symptoms on day-to-day activities, such as getting dressed or doing housework, and B) any changes in cognitive abilities. Respondents are asked to rate the degree of difficulty performing various activities or cognitive tasks, including concentration and memory, over the past 7 days. Daily activities items were rated on a 5-point scale from “without any difficulty” to “with much difficulty” and included the option “unable to do”; cognitive ability items were rated on a 5-point scale from “not at all” to “very much”.

**References (literature review)**

1. Herr, J. K., Salyer, J., Lyon, D. E., Goodloe, L., Schubert, C., & Clement, D. G. (2014). Heart failure symptom relationships: a systematic review. *J Cardiovasc Nurs, 29*(5), 416-422.

2. Bekelman, D. B., Havranek, E. P., Becker, D. M., Kutner, J. S., Peterson, P. N., Wittstein, I. S., et al. (2007). Symptoms, depression, and quality of life in patients with heart failure. *J Card Fail, 13*(8), 643-648.

3. Albert, N., Trochelman, K., Li, J., & Lin, S. (2010). Signs and symptoms of heart failure: are you asking the right questions? *Am J Crit Care, 19*(5), 443-452.

4. Aldred, H., Gott, M., & Gariballa, S. (2005). Advanced heart failure: impact on older patients and informal carers. *J Adv Nurs, 49*(2), 116-124.

5. Ambrosy, A. P., Pang, P. S., Khan, S., Konstam, M. A., Fonarow, G. C., Traver, B., et al. (2013). Clinical course and predictive value of congestion during hospitalization in patients admitted for worsening signs and symptoms of heart failure with reduced ejection fraction: findings from the EVEREST trial. *Eur Heart J, 34*(11), 835-843.

6. Avery, C. L., Mills, K. T., Chambless, L. E., Chang, P. P., Folsom, A. R., Mosley, T. H., et al. (2010). Long-term association between self-reported signs and symptoms and heart failure hospitalizations: the Atherosclerosis Risk In Communities (ARIC) Study. *Eur J Heart Fail, 12*(3), 232-238.

7. Biagi, P., Gussoni, G., Iori, I., Nardi, R., Mathieu, G., Mazzone, A., et al. (2011). Clinical profile and predictors of in-hospital outcome in patients with heart failure: the FADOI "CONFINE" Study. *Int J Cardiol, 152*(1), 88-94.

8. Blinderman, C. D., Homel, P., Billings, J. A., Portenoy, R. K., & Tennstedt, S. L. (2008). Symptom distress and quality of life in patients with advanced congestive heart failure. *J Pain Symptom Manage, 35*(6), 594-603.

9. Calvert, M. J., Freemantle, N., & Cleland, J. G. (2005). The impact of chronic heart failure on health-related quality of life data acquired in the baseline phase of the CARE-HF study. *Eur J Heart Fail, 7*(2), 243-251.

10. Costello, J. A., & Boblin, S. (2004). What is the experience of men and women with congestive heart failure? *Can J Cardiovasc Nurs, 14*(3), 9-20.

11. Edmonds, P. M., Rogers, A., Addington-Hall, J. M., McCoy, A., Coats, A. J., & Gibbs, J. S. (2005). Patient descriptions of breathlessness in heart failure. *Int J Cardiol, 98*(1), 61-66.

12. Ekman, I., Cleland, J. G., Swedberg, K., Charlesworth, A., Metra, M., & Poole-Wilson, P. A. (2005). Symptoms in patients with heart failure are prognostic predictors: insights from COMET. *J Card Fail, 11*(4), 288-292.

13. Friedman, M. M., & Quinn, J. R. (2008). Heart failure patients' time, symptoms, and actions before a hospital admission. *J Cardiovasc Nurs, 23*(6), 506-512.

14. Gwaltney, C. J., Slagle, A. F., Martin, M., Ariely, R., & Brede, Y. (2012). Hearing the voice of the heart failure patient: key experiences identified in qualitative interviews. *Br J Cardiol, 19*, 25.

15. Johansson, P., Nieuwenhuis, M., Lesman-Leegte, I., van Veldhuisen, D. J., & Jaarsma, T. (2011). Depression and the delay between symptom onset and hospitalization in heart failure patients. *Eur J Heart Fail, 13*(2), 214-219.

16. Jones, J., McDermott, C. M., Nowels, C. T., Matlock, D. D., & Bekelman, D. B. (2012). The experience of fatigue as a distressing symptom of heart failure. *Heart Lung, 41*(5), 484-491.

17. Jurgens, C. Y. (2006). Somatic awareness, uncertainty, and delay in care-seeking in acute heart failure. *Res Nurs Health, 29*(2), 74-86.

18. Kato, M., Stevenson, L. W., Palardy, M., Campbell, P. M., May, C. W., Lakdawala, N. K., et al. (2012). The worst symptom as defined by patients during heart failure hospitalization: implications for response to therapy. *J Card Fail, 18*(7), 524-533.

19. Lee, K. S., Song, E. K., Lennie, T. A., Frazier, S. K., Chung, M. L., Heo, S., et al. (2010). Symptom clusters in men and women with heart failure and their impact on cardiac event-free survival. *J Cardiovasc Nurs, 25*(4), 263-272.

20. Moser, D. K., Yamokoski, L., Sun, J. L., Conway, G. A., Hartman, K. A., Graziano, J. A., et al. (2009). Improvement in health-related quality of life after hospitalization predicts event-free survival in patients with advanced heart failure. *J Card Fail, 15*(9), 763-769.

21. Opasich, C., Gualco, A., De Feo, S., Barbieri, M., Cioffi, G., Giardini, A., et al. (2008). Physical and emotional symptom burden of patients with end-stage heart failure: what to measure, how and why. *J Cardiovasc Med (Hagerstown), 9*(11), 1104-1108.

22. Parshall, M. B., Carle, A. C., Ice, U., Taylor, R., & Powers, J. (2012). Validation of a three-factor measurement model of dyspnea in hospitalized adults with heart failure. *Heart Lung, 41*(1), 44-56.

23. Rector, T. S., Anand, I. S., & Cohn, J. N. (2006). Relationships between clinical assessments and patients' perceptions of the effects of heart failure on their quality of life. *J Card Fail, 12*(2), 87-92.

24. Rodriguez, K. L., Appelt, C. J., Switzer, G. E., Sonel, A. F., & Arnold, R. M. (2008). "They diagnosed bad heart": a qualitative exploration of patients' knowledge about and experiences with heart failure. *Heart Lung, 37*(4), 257-265.

25. Shah, A. B., Udeoji, D. U., Baraghoush, A., Bharadwaj, P., Yennurajalingam, S., & Schwarz, E. R. (2013). An evaluation of the prevalence and severity of pain and other symptoms in acute decompensated heart failure. *J Palliat Med, 16*(1), 87-90.

26. Smithline, H. A., Caglar, S., & Blank, F. S. (2010). Physician vs patient assessment of dyspnea during acute decompensated heart failure. *Congest Heart Fail, 16*(2), 60-64.

27. Son, Y. J., Lee, Y., & Song, E. K. (2011). Adherence to a sodium-restricted diet is associated with lower symptom burden and longer cardiac event-free survival in patients with heart failure. *J Clin Nurs, 20*(21-22), 3029-3038.

28. Song, E. K., Moser, D. K., Rayens, M. K., & Lennie, T. A. (2010). Symptom clusters predict event-free survival in patients with heart failure. *J Cardiovasc Nurs, 25*(4), 284-291.

29. Zaharias, E., Cataldo, J., Mackin, L., & Howie-Esquivel, J. (2014). Simple measures of function and symptoms in hospitalized heart failure patients predict short-term cardiac event-free survival. *Nurs Res Pract, 2014*, 815984.

30. Zambroski, C. H., Moser, D. K., Bhat, G., & Ziegler, C. (2005). Impact of symptom prevalence and symptom burden on quality of life in patients with heart failure. *Eur J Cardiovasc Nurs, 4*(3), 198-206.

31. Dekker, R. L., Peden, A. R., Lennie, T. A., Schooler, M. P., & Moser, D. K. (2009). Living with depressive symptoms: patients with heart failure. *Am J Crit Care, 18*(4), 310-318.

32. Maurer, M. S., Cuddihy, P., Weisenberg, J., Delisle, S., Strong, B. M., Gao, Q., et al. (2009). The prevalence and impact of anergia (lack of energy) in subjects with heart failure and its associations with actigraphy. *J Card Fail, 15*(2), 145-151.

33. Kindermann, I., Fischer, D., Karbach, J., Link, A., Walenta, K., Barth, C., et al. (2012). Cognitive function in patients with decompensated heart failure: the Cognitive Impairment in Heart Failure (CogImpair-HF) study. *Eur J Heart Fail, 14*(4), 404-413.

34. Pressler, S. J., Subramanian, U., Kareken, D., Perkins, S. M., Gradus-Pizlo, I., Sauve, M. J., et al. (2010). Cognitive deficits and health-related quality of life in chronic heart failure. *J Cardiovasc Nurs, 25*(3), 189-198.

35. Schowalter, M., Gelbrich, G., Stork, S., Langguth, J. P., Morbach, C., Ertl, G., et al. (2013). Generic and disease-specific health-related quality of life in patients with chronic systolic heart failure: impact of depression. *Clin Res Cardiol, 102*(4), 269-278.

36. Xiong, G. L., Fiuzat, M., Kuchibhatla, M., Krishnan, R., O'Connor, C. M., Jiang, W., et al. (2012). Health status and depression remission in patients with chronic heart failure: patient-reported outcomes from the SADHART-CHF trial. *Circ Heart Fail, 5*(6), 688-692.

37. Brostrom, A., Stromberg, A., Dahlstrom, U., & Fridlund, B. (2004). Sleep difficulties, daytime sleepiness, and health-related quality of life in patients with chronic heart failure. *J Cardiovasc Nurs, 19*(4), 234-242.

38. Moser, D. K., Frazier, S. K., Worrall-Carter, L., Biddle, M. J., Chung, M. L., Lee, K. S., et al. (2011). Symptom variability, not severity, predicts rehospitalization and mortality in patients with heart failure. *Eur J Cardiovasc Nurs, 10*(2), 124-129.

39. Yamada, S., Shimizu, Y., Suzuki, M., Izumi, T., & collaborators, P. T. (2012). Functional limitations predict the risk of rehospitalization among patients with chronic heart failure. *Circ J, 76*(7), 1654-1661.

40. Kosiborod, M., Krumholz, H. M., Jones, P. G., Pitt, B., & Spertus, J. A. (2008). The relationship between anemia, change in hematocrit over time and change in health status in patients with heart failure after myocardial infarction. *J Card Fail, 14*(1), 27-34.

# Supplementary Appendix 1 – CE interview guide

**Introduction**

- Thank you for agreeing to participate in this study and taking the time to speak with me today.
- In this study, we’re interested in learning about what it’s like to have heart failure and how it impacts your life, especially with respect to your heart failure symptoms.
- You will receive a $125 Visa gift card at the end of the interview. The interview will last approximately one 60 minutes.
- All of the information you provide will be used for the purpose of this research project only. You do not have to answer any questions you don’t want to, and you are free to stop the interview at any time. If you would like to stop, just let me know.
- You are also welcome to take a break at any time. If you need a break, please just let me know.
- There are no right or wrong answers to these questions. We are interested in your thoughts and opinions.
- My role here is to ask questions and to listen.  I am not a medical expert, so I will not be able to answer any questions about heart failure.  I’m here to learn from you about your experiences. If you have a question about your heart failure or any other health concern, please contact your medical care provider.
- With your permission, I will be audio-recording this interview, and the audio-recording will be transcribed to allow us to review your responses.
- All of your comments will be kept confidential, and your name will not be included in the audio-recording, the interview transcript or any reports or publications that may result from this study Instead, we will use a participant number to identify you.
- Do you have any questions before we get started?
- Before we begin the interview, I will ask you to complete a brief background questionnaire [Hand participant the socio-demographic form or complete it with them via phone. *Put July 01 as default birth date and only collect the year per ICON SOP.]*
- We are now ready to proceed with the interview. Do I have your permission to audio-record the interview?

[If NO] As part of the study, we are required to audio-record the interviews so that we can analyze the responses to our questions for this research study. If you do not agree to be audio-recorded, you will not be able to participate in this study. We will protect your confidentiality by not associating your name with any of your comments. Do you agree to be audio-recorded?

[*If participant declines to be audio-recorded, thank the participant for their time and terminate interview.]*

- Thank you. Now I will turn on the audio-recorder. First, I will say the name of the study, followed by the date and then your study ID number. I will not say your name on the recording.

*Start audio-recorder.*

- The recorder is now on. This is study 0018-0632, today is [date], and this is participant [ID]
- To confirm, do I have your permission to record the interview?
- Now we’re ready to get started.

**Opening Questions**

To start, I’m very interested to learn from you about what it’s like to have heart failure.

1. What is it like to have heart failure?
2. How long have you had heart failure?
3. How did you find out you had heart failure?
   1. Changes in how you felt?
   2. Changes in what you could do?

**Current Symptoms**

Now I’d like to talk about your present symptoms.

1. Which symptoms have you experience?

*Ask the following questions for each symptom mentioned:*

| 1. **What is X like? How would you describe X?** 2. **Do you believe X is from the heart failure or something else? (If other, probe: another health problem? A medication or treatment you are receiving?)** 3. **How often do you have X? Does the frequency change from day to day?** 4. **How long does X last? Does that change from day-to-day or throughout the day?** 5. **How severe is X? (Is it more severe than other symptoms?) Using a scale where “0 is not bad at all” and “10 is as bad as it gets,” how would you rate it?   Does the severity change from day to day or throughout the day?   Has it changed over time?** 6. **How bothersome do you find X?**     1. **Using a scale where 0 is not at all bothersome to 10 is extremely bothersome, how would you rate it?**    2. **What about X makes it bothersome?** 7. **What is it like when X is at its worst? How often does that happen?** 8. **What is it like when it’s at its mildest? How often does that happen?** 9. **How does X affect your everyday life?**     - **Can you give me an example? *(Getting showered? Getting dressed? Household chores? Going outside the house? Work? Running errands? Recreation?)***    - ***Does X affect your plans? Your social life? Relationships? Mood?***   ***[IF YES: can you please describe to me how X affects your [plans, social life, relationships]?***   - - **Any other ways that X affects your everyday life?** |
| --- |

*If the following symptoms (#5-9) were not spontaneously mentioned, continue to ask questions A-I (in the box below) for each symptom the patient experiences.*

1. Do you ever experience **breathing problems**?

*Even if the respondent states they do not have breathing problems, ask the following:*

- Does your breathing get worse when you lay down?
- Do breathing problems wake you up at night?
- How long can you walk before you are out of breath?

*Complete the dyspnoea scale now or wait until the end of the interview.*

1. Do you ever experience **edema,** or **swelling** in your arms/legs or other parts of your body?

IF YES, where do you have swelling?
IF YES, do you take medication to alleviate the swelling? How much swelling does it alleviate?

*Even if the respondent states they do not experience edema or swelling etc, ask the following:*

- Do you experience sudden weight gain?
- Are there days when it is more difficult to fit into your clothes, shoes or jewellery such as rings or watches?

1. Do you experience **fatigue, lack of energy, or tiredness**?

*Even if the respondent states they do not experience fatigue etc, ask the following:*

- Do you feel refreshed and energized when you wake up in the morning?
- Do you nap during the day? [IF YES: Do you nap every day? How many naps do you typically take on a normal day? Do you feel refreshed and re-energized when you wake up from a nap?]

1. Do you experience **chest pain?**
2. Do you experience **pain** in any other part of your body?
   1. IF YES: Where do you have pain?

| 1. **What is X like? How would you describe X?** 2. **Do you believe X is from the heart failure or something else? (If other, probe: another health problem? A medication or treatment you are receiving?)** 3. **How often do you have X? Does it the frequency change from day to day?** 4. **How long does X last? Does that change from day-to-day or throughout the day?** 5. **How severe is X? (Is it more severe than other symptoms?) Using a scale where 0 is “not at all” and 10 is “as bad as it gets” how would you rate it?  Does the severity change from day to day or throughout the day?  Has it changed over time?** 6. **How bothersome do you find X?**     1. **Using a scale where 0 is not at all bothersome and 10 is extremely bothersome, how would you rate it?**    2. **What about X makes it bothersome?** 7. **What is it like when X is at its worst? How often does that happen?** 8. **What is it like when it’s at its mildest? How often does that happen?** 9. **How does X affect your everyday life?**     - **Can you give me an example? *(Getting showered? Getting dressed? Household chores? Going outside the house? Work? Running errands?)***    - **Does X affect your plans? Your social life? Relationships?**   ***IF YES: can you please describe to me how [symptom] affects your [plans, social life, relationships]?***   - - **Any other ways that X affects your everyday life?** |
| --- |

*If not mentioned spontaneously, ask:*

1. Do you ever notice any changes in your **mood or thinking** (if need clarification for thinking: includes concentration, memory, confusion)? If so, do you believe this is related to your heart failure?

*Do you ever feel…Depressed or sad? Anxious or nervous? Irritable? Restless? Frustrated?*

*Ask about each of these symptoms separately*

- 1. Can you tell me what that is like?
  2. How does this affect your life?
  3. Does this mood come and go or is it constant?
  4. Have you received any treatment for this?
     1. IF YES: can you please describe the treatment you receive (d) for your [mood symptom]? Does the treatment help?]

*Do you have problems concentrating? Do you have trouble remembering things? Do you ever feel unable to think clearly?*

- 1. Can you tell me what that is like?
  2. How does this affect your life?
  3. Does this thinking problem come and go or is it constant? Have you received any treatment for this?
  4. IF YES: can you please describe the treatment you receive (d) for your [cognitive issue]? Does the treatment help?

**Questions for Stable Patients**

1. In addition to the symptoms you’ve experienced in the past week, are there any other symptoms you think are related to your heart failure that you have experienced?
   1. IF YES (for each symptom mentioned):
      1. Can you please describe [symptom]?
      2. When was the last time you experienced [symptom]?
      3. How often do you experience this symptom?
   2. Has [symptom] changed over time?

**Questions for ADHF Patients**

Now I’d like for you to think about your most recent hospitalization for heart failure.

1. What led to your hospitalization?
   1. Did you experience a change in how you were feeling?
   2. What was that change?  *Did a symptom get worse? How did it get worse? More severe? More frequent? Lasted a longer time? Any other ways the [symptom] changed? Did you experience a new symptom? Were you less able or unable to do something you can normally do?*
   3. How long was it from the time you started to experience these symptom changes to the time you went to see the doctor or went to the hospital?
   4. Did [symptom] get worse over this time?
   5. What was it that made you go to the doctor or hospital about your [symptom]?
   6. At the time you were admitted to the hospital for your heart failure, on a scale of 1 – 10 where 1 equals not at all bad and 10 equals as bad as it ever gets, how bad was [symptom]?
      1. Can you describe what that felt like?
2. At the time you left the hospital, what were your symptoms like?
   1. On a scale of 1 – 10 where 1 equals not at all bad and 10 equals as bad as it ever gets, how bad was [symptom] on the day you were discharged from the hospital?
3. Did [symptom] continue to improve *after* you left the hospital?
   1. IF YES: on a scale of 1 – 10 where 1 equals not at all bad and 10 equals as bad as it ever gets, what is your [symptom] like now?

*If there is a difference in symptom rating from time of discharge, probe:*

- - 1. About how long after you left the hospital did you notice a change in your [symptom]?
    2. In what way did your [symptom] change? Did it become less severe? Less bothersome? Less frequent? Did it last for less time?
    3. How did the change in [symptom] affect your life?

1. Compared to how you were before your HF symptoms worsened and you were admitted to the hospital, are you now:
   1. About the same as you were? A little worse? A lot worse? A little better? A lot better?
      *Can you tell me why you think you are [selected option]?*
   2. Have you changed your everyday activities to accommodate your symptoms?
   3. Do you need more/less help than you did before your most recent HF hospitalization?
   4. Do you feel physically: the same? A little worse? A lot worse? A little better? A lot better?
   5. Do you feel emotionally: the same? A little worse? A lot worse? A little better? A lot better?
   6. Is your ability to think (including concentration, memory, clarity): the same? A little worse? A lot worse? A little better? A lot better?

**Impacts**

1. In general, how does having heart failure affect your everyday life?
   *If the following impacts were not already mentioned, ask about:*
   1. Does heart failure affect what you can do inside the home? What about activities outside the home?
   2. Does heart failure ever prevent you from leaving the house?
   3. Has heart failure affected your sleep quality, or your sleep habits? (Probes: do you need to sleep inclined or use extra pillows?)
   4. Ability to exercise or engage in physical activities?
   5. Ability to maintain close relationships, including physical intimacy?
   6. Has heart failure affected you financially in any way?
   7. Do you worry about what will happen in the future?
   8. What bothers you the most about your heart failure?
2. Do you have any help taking care of yourself or keeping up with chores or errands?
   1. If YES: What do you need help with? Do you always need help with this or just sometimes? IF SOMETIMES: When do you need help with this? Who helps you? How often do you need
3. Is there something that you would like help with that you don’t have help with now?
4. If YES: What do you need help with? Do you always need help with this or just sometimes? IF SOMETIMES: When do you need help with this?
5. How does needing help with [activity/function] make you feel?

**Dyspnoea Scale**

*Prior to completion of each interview, ask the following:*

18. Now I would like to ask you to rate your breathing. I am going to read you some statements about breathing. Please tell me which statement best fits you *right now*.

| **Grade** | **Degree of breathlessness related to activities** |
| --- | --- |
| 1 | Not troubled by breathlessness except on strenuous exercise |
| 2 | Short of breath when hurrying on the level or walking up a slight hill |
| 3 | Walks slower than most people on the level, stops after a mile or so, or stops after 15 minutes of walking at own pace |
| 4 | Stops for breath after walking about 100 yards or after a few minutes on level ground |
| 5 | Too breathless to leave the house, or breathless when undressing |

*Alternate wording:*

*1. Not troubled by breathlessness except during strenuous exercise*

*2. Short of breath when hurrying on flat ground or walking up a slight hill*

*3. Walk slower than most people on flat ground, and stop after a mile or so, or stop after 15 minutes of walking at normal pace.*

*4. Stop for breath after walking about 100 yards (length of football field) or a few minutes on flat ground*

*5. Too breathless to leave the house, or breathless when undressing*

**Conclusion**

1. Are there any other symptoms you experience that we haven’t already talked about?
   *If YES, repeat symptom-specific questions (A-I in the box) for each symptom.*
2. Is there anything that you would like to share regarding your heart failure?
3. Is there anything that you think we have left out or did not discuss in this interview?

Thank you for taking the time to speak with me today. This completes the interview.

[Turn off the audio-recorder.]

***[For phone interviews, confirm the participant’s mailing address for the gift card after the audio recording is turned off.]***

**Supplementary Table 1. Gap analysis grid**

|  | N  (CE Study) | Lit  Review | KOLs | EHFScBS-9 | HeartQoL | KCCQ | MacNew | MLHFQ | CAMPHOR | DSS | MSAS-HF | PMADL-8 |
| --- | --- | --- | --- | --- | --- | --- | --- | --- | --- | --- | --- | --- |
| Number of Items---> |  |  |  | 9 | 14 | 23 | 27 | 21 | 65 | 4 | 96 | 8 |
| Recall period |  |  |  | Contemporaneous | 2 weeks | 1 week | 2 weeks | 1 month | Contemporaneous | Daily | 1 week | 1 month |
| **PHYSIOLOGICAL SYMPTOMS** | |  |  |  |  |  |  |  |  |  |  |  |
| SOB/dyspnoea | 25 | x | x | xx | x | xx | x | x | x | x | xxxxx xxxx |  |
| Oedema | 23 | x | x | xxx |  | xx |  | x |  | x | xxx |  |
| Fatigue | 22 | x | x | xx | x | xx | x | xx | x | x | xxx |  |
| Weakness | 6 | x |  |  |  |  |  |  | x |  |  |  |
| Chest pain | 5 | x | x |  |  |  | x |  |  |  | xxx |  |
| Light headed/dizzy | 4 | x | x |  |  |  | x |  |  |  | xxx |  |
| Heart palpitations | 4 | x | x |  |  |  |  |  |  |  | xxx |  |
| Cough | 3 | x | x |  |  |  |  |  |  |  | xxx |  |
| **COGNITIVE SYMPTOMS** | | x | x |  |  |  |  |  |  |  |  |  |
| Memory | 11 | x |  |  |  |  |  | x |  |  |  |  |
| Concentration | 9 | x |  |  |  |  |  | x |  |  | xxx |  |
| **Physical Functioning** |  |  |  |  |  |  |  |  |  |  |  |  |
| Walking | 25 | x | x |  | x | x |  | x | x |  |  | xx |
| Sleep | 12 | x | x |  |  |  |  | x |  |  | xxx |  |
| Stairs | 12 | x | x |  | x | x |  | x | x | x |  | x |
| Lifting/carrying | 11 | x | x |  | x |  |  |  | x |  |  | x |
| **Daily activities** |  |  | x |  |  |  |  |  |  |  |  |  |
| Errands/shopping | 12 | x |  |  |  |  |  | x |  |  |  |  |
| Indoor chores | 11 | x |  |  |  | x |  |  |  |  |  | x |
| Staying at home | 5 | x | x |  |  |  |  |  |  |  |  |  |
| **Self-care activities** |  | x | x |  |  |  |  |  |  |  |  |  |
| Shower/bathe | 6 |  |  |  |  |  |  |  |  |  |  | x |
| Dressing | 4 |  |  |  |  | x |  |  | x |  |  |  |

| **OTHER CONCEPTS REPORTED IN STUDY** | | | |  |  |  |  |  |  |  |  |  |
| --- | --- | --- | --- | --- | --- | --- | --- | --- | --- | --- | --- | --- |
| Financial | 13 | x |  |  |  |  |  | x |  |  |  |  |
| Changed diet | 7 | x |  | x |  | x |  | x |  |  |  |  |
| Yard work | 5 | x |  |  | x | x |  | x |  |  |  |  |
| Nausea | 2 | x | x |  |  |  |  |  |  |  | xxx |  |
| **Emotional symptoms** | | |  |  |  |  |  |  | xxxxx |  |  |  |
| Worry about future | 14 | x | x |  | x |  |  | x |  |  | xxx |  |
| Frustration | 10 | x |  |  | x |  | x |  |  |  |  |  |
| Depression | 7 | x | x |  | x | xxx | xxx | x | x |  | xxx |  |
| Irritability | 6 | x | x |  |  |  |  |  |  |  | xxx |  |
| Fear | 6 | x |  |  |  |  | x |  |  |  |  |  |
| Anxiety/nervousness | 4 | x | x |  |  |  |  |  | x |  | xxx |  |
| Weakness |  |  |  |  |  |  |  |  |  |  |  |  |
| **Exercise/fitness** | 13 | x |  | x | x |  | xx | x |  |  |  |  |
| **Recreation** | 13 | x |  |  |  |  | x | x |  |  |  |  |
| **Employment** | 12 | x |  |  |  |  |  | x |  |  |  |  |
| **Social impacts** |  |  |  |  |  |  |  |  |  |  |  |  |
| Sexual dysfunction | 13 | x |  |  |  | x |  | x |  |  | xxx |  |
| Social outings | 7 | x | x |  |  | x | xxx | x | x |  |  |  |
| Difficulty talking | | |  |  |  |  |  |  |  |  |  |  |
| **Other concepts not  included in conceptual  model** | | |  | -Weigh self daily  -Take medication as prescribed  -Get annual  flu shot |  | -Difficulty hurrying | -Feel like you’re not as good as others  -Feel dependent on others  -Free of tension  -Others have lost confidence in you  -Family is overprotective  -Pleased with life  -Lack self-confidence  -Unsure of appropriate activity level  -Aching legs | -Loss of self-control in your life  -Stay in the hospital  -Treatment side effects |  |  | -Loss of Appetite  -Dry mouth  -Drowsiness  -Vomiting | -Getting up from the floor  -Getting in/out of car |

CAMPHOR, Cambridge Pulmonary Hypertension Outcome Review questionnaire; CE, concept elicitation; DSS, Daily Symptom Score; EHFScBS-9, European Heart Failure Self-care Behaviour Scale, 9 items; HeartQoL, Heart Quality of Life; KCCQ, Kansas City Cardiomyopathy Questionnaire; KOL, key opinion leader; MacNew, MacNew Heart Disease Health-related Quality of Life Questionnaire; MLHFQ, Minnesota Living with Heart Failure Questionnaire; MSAS-HF, Memorial Symptom Assessment Scale for Heart Failure; PMADL-8, Performance Measure for Activities of Daily Living-8; SOB, shortness of breath.

NOTE: The number of x’s reflects the number of items specific to a concept included in the measure

# Supplementary Table 2. Summary of item revisions in the HF-DSD across the three waves of cognitive interviews

| Original Items | Recommended changes and rationale (Wave 1 to 2) | Wave 2 Version | Recommended changes and rationale (Wave 2 to 3) | Wave 3 Version | Recommended changes and rationale (Wave 3 to final) |
| --- | --- | --- | --- | --- | --- |
| **Instrument Instructions**  [*No general instructions in Wave 1 instrument*] | General instructions used rather than symptom-specific instructions | **Instructions**  **Please select the number that best reflects your experience of each symptom in the past 24 hours.** |  | **Instructions**  Please answer the following questions based on how you experience symptoms related to **heart failure**. If you have other conditions that affect these symptoms, **please try to answer based only on heart failure.** Please select the number that best reflects your experience of each symptom **in the past 24 hours.** | No changes |
| **SOB – Instructions**  Please select the number that best reflects your experience of shortness of breath in the past 24 hours. | Removed; no longer necessary after symptom questions were consolidated. |  |  |  |  |
| **SOB – Frequency**  1. In the past 24 hours, how frequently did you experience shortness of breath?  0 = I had no shortness of breath  10 = I was always short of breath | When combined, only 4/11 participants correctly interpreted all three dimensions of Shortness of Breath.  6/11 confused either frequency or duration for severity.  1 considered severity to be a matter of frequency. | **Shortness of Breath**  1. In the past 24 hours, how bad was your **shortness of breath** at **its worst?**  0 = I had no shortness of breath  10 = It felt very severe | No changes |  |  |
| **SOB – Severity**  2. In the past 24 hours, how bad was your shortness of breath at its worst?  0 = It felt very mild  10 = It felt very severe |  |  |  |  |  |
| **SOB – Duration**  3. In the past 24 hours, how long did your shortness of breath last?  0 = It lasted only for a moment  10 = It lasted for a very long time |  |  |  |  |  |
| **Orthopnoea**  4. In the past 24 hours, how bad was your shortness of breath when lying flat?  0 = When I lay down flat, I had no shortness of breath  10 = I cannot lay down flat at all because of my shortness of breath | No changes made. | **Orthopnoea**  2. In the past 24 hours, how bad was your **shortness of breath when lying flat**?  0 = When I lay down flat, I had no shortness of breath  10 = I cannot lay down flat at all because of my shortness of breath | Modification of anchors based on translatability assessment. | **Orthopnoea**  2. In the past 24 hours, how bad was your **shortness of breath when lying flat?**  0 = When I was lying down flat, I had no shortness of breath  10 = I could not lay down flat at all because of my shortness of breath | No changes |
| **Chest pain – Instructions**  Please select the number that best reflects your experience of **chest pain** in the **past 24 hours**. | Not necessary when symptom questions are consolidated. |  |  |  |  |
| **Chest Pain – Frequency**  5. In the past 24 hours, how frequently did you experience **chest pain**?  0 = I never had chest pain  10 = I always had chest pain | When combined, only 6/11 participants correctly interpreted all three dimensions of Chest Pain.  5/11 confused the meanings of the three dimensions. | **Chest pain**  3. In the past 24 hours, how bad was your **chest pain** at **its worst?**  0 = I had no chest pain  10 = It felt very severe | No changes |  |  |
| **Chest Pain – Severity**  6. In the past 24 hours how bad was your **chest pain** at **its worst?**  0 = It felt very mild  10 = It felt very severe |  |  |  |  |  |
| **Chest Pain – Duration**  7. In the past 24 hours, how long did your **chest pain** last?  0 = It lasted only for a moment  10 = It lasted for a very long time |  |  |  |  |  |
| **Cough – Instructions**  Please select the number that best reflects your experience of **coughing** in the **past 24 hours**. | Not necessary when symptom questions are consolidated. |  |  |  |  |
| **Cough – Frequency**  8. In the past 24 hours, how frequently did you **cough**?  0 = I never had cough  10 = I always had cough | When combined, only 6/11 participants correctly interpreted all three dimensions of Cough.  5/11 confused the meanings of the three dimensions. | **Cough**  4. In the past 24 hours, how bad was your **cough** at **its worst?**  0 = I did not have cough  10 = It felt very severe | No changes |  |  |
| **Cough – Severity**  9. In the past 24 hours how bad was your **cough** at **its worst**?  0 = It felt very mild  10 = It felt very severe |  |  |  |  |  |
| **Cough – Duration**  10. In the past 24 hours, how long did your **coughing** last?  0 = It lasted only for a moment  10 = It lasted for a very long time |  |  |  |  |  |
| **Wheezing – Instructions**  Please select the number that best reflects your experience of **wheezing** in the **past 24 hours**. | Not necessary when symptom questions are consolidated. |  |  |  |  |
| **Wheezing – Frequency**  11. In the past 24 hours, how frequently did you **wheeze**?  0 = I never wheezed  10 = I always wheezed | When combined, only 5/11 participants correctly interpreted all three dimensions of Wheezing.  3/11 confused the meanings of the three dimensions.  3/11 indicated that they did not understand the term “wheezing.” | **Wheezing**  5. In the past 24 hours, how bad was your **wheezing** at **its worst?**  0 = I did not wheeze  10 = It felt very severe | No changes |  |  |
| **Wheezing – Severity**  12. In the past 24 hours how bad was your **wheezing** at **its worst?**  0 = It felt very mild  10 = It felt very severe |  |  |  |  |  |
| **Wheezing – Duration**  13. In the past 24 hours, how long did your **wheezing** last?  0 = It lasted only for a moment  10 = It lasted for a very long time |  |  |  |  |  |
| **Swelling – Instructions**  Please select the number that best reflects your **swelling in the feet or ankles** in the **past 24 hours**. | Not necessary when symptom questions are consolidated. |  |  |  |  |
| **Swelling – Frequency**  14. In the past 24 hours, how frequently did you experience **swelling in your feet/ankles**?  0 = I never had swelling  10 = I always had swelling | When combined, only 2/11 participants correctly interpreted all three dimensions of Swelling.  9/11 confused the meanings of the three dimensions, reporting answers to frequency as being determined by either duration or severity. | **Oedema**  6. In the past 24 hours, how bad was your **swelling in your feet/ankles** at **its worst?**  0 = I did not have swelling  10 = It felt very severe | No changes |  |  |
| **Swelling – Severity**  15. In the past 24 hours how bad was the **swelling in your feet/ankles** at **its worst?**  0 = It felt very mild  10 = It felt very severe |  |  |  |  |  |
| **Swelling – Duration**  16. In the past 24 hours, how long did the **swelling in your feet or ankles** last?  0 = It lasted only for a moment  10 = It lasted for a very long time |  |  |  |  |  |
| **Tiredness – Instructions**  Please select the number that best reflects your **tiredness (or sluggishness)** in the **past 24 hours.** | Not necessary when symptom questions are consolidated. |  |  |  |  |
| **Tiredness – Frequency**  17. In the past 24 hours, how frequently did you experience **tiredness**?  0 = I was not at all tired  10 = I was always tired | When combined, only 3/11 participants correctly interpreted the dimensions of Tiredness.  8/11 confused the meanings of the two dimensions, reporting answers to frequency as being determined by severity. | **Tiredness**  7. In the past 24 hours, how bad was your **tiredness** at **its worst?**  0 = I was not tired  10 = It felt very severe | No changes |  |  |
| **Tiredness – Severity**  18. In the past 24 hours how bad was the **tiredness** at **its worst**?  0 = It felt very mild  10 = It felt very severe |  |  |  |  |  |
| **Weakness – Instructions**  Please select the number that best reflects your experience with **weakness (or lack of strength)** in the **past 24 hours**. | Not necessary when symptom questions are consolidated. |  |  |  |  |
| **Weakness – Frequency**  19. In the past 24 hours, how frequently did you experience **weakness or a lack of strength**?  0 = I never felt weak  10 = I always felt weak | When combined, only 6/11 participants correctly interpreted dimensions of Weakness.  5/11 confused the meanings of the two dimensions. 4 reported Frequency as Severity, and 2 reported Severity as Frequency (1 patient swapped the response scales). | **Weakness**  8. In the past 24 hours, how bad was your **weakness (lack of strength)** at **its worst?**  0 = I did not feel weak  10 = It felt very severe | Addition of the term of “physical” to the item wording for clarity, to help distinguish from the tiredness item. | 8. In the past 24 hours, how bad was your **weakness (lack of physical strength)** at **its worst?**  0 = I did not feel weak  10 = It felt very severe | No changes |
| **Weakness – Severity**  20. In the past 24 hours how bad was the **weakness or a lack of strength** at **its worst**?  0 = It felt very mild  10 = It felt very severe |  |  |  |  |  |
| **Dizziness – Instructions**  Please select the number that best reflects your experience with **dizziness (or feeling light-headed)** in the **past 24 hours**. | Not necessary when symptom questions are consolidated. |  |  |  |  |
| **Dizziness – Frequency**  21. In the past 24 hours, how frequently did you experience **dizziness (or feeling light-headed)?**  0 = I never felt dizzy  10 = I very frequently felt dizzy | When combined, only 5/11 participants correctly interpreted dimensions of Dizziness.  3/11 confused the meanings of the dimensions.  3/11 provided answers that indicated they did not correctly interpret dizziness or light-headedness. | **Dizziness**  9. In the past 24 hours, how bad was your **dizziness (or feeling light-headed)** at **its worst?**  0 = I did not feel dizzy  10 = It felt so dizzy that I was unable to stand | No changes |  |  |
| **Dizziness – Severity**  22. In the past 24 hours how bad was the **dizziness (or feeling light-headed)** at **its worst?**  0 = It felt very mild  10 = It felt so dizzy that I was unable to stand |  |  |  |  |  |
| **Palpitations – Instructions**  Please select the number that best reflects your experience with **heart palpitations** in the **past 24 hours**. | Not necessary when symptom questions are consolidated. |  |  |  |  |
| **Palpitations – Frequency**  23. In the past 24 hours, how frequently did you experience **heart palpitations?**  0 = I never had heart palpitations  10 = I very frequently had heart palpitations | When combined, only 4/11 participants correctly interpreted the three dimensions of Palpitations.  3/11 confused the meanings of the frequency and severity.  2/11 indicated they did not know what palpitations are.  2/11 indicated that there is no difference between duration and severity. | **Palpitations**  10. In the past 24 hours, how bad were your **heart palpitations** (pounding, fluttering, or fast heart-beat) at **their worst?**  0 = I did not have heart palpitations  10 = They felt very severe | Minor grammatical change per suggestion of one patient: added an “or” before “fluttering.” | **Palpitations**  10. In the past 24 hours, how bad were your **heart palpitations** (pounding, or fluttering, or fast heart-beat) at **their worst?**  0 = I did not have heart palpitations  10 = They felt very severe | No changes |
| **Palpitations – Severity**  24. In the past 24 hours how bad were your **heart palpitations** at **their worst**?  0 = They felt very mild  10 = They felt very severe |  |  |  |  |  |
| **Palpitations – Duration**  25. In the past 24 hours, how long did your **heart palpitations** last?  0 = They lasted only for a moment  10 = They lasted for a very long time |  |  |  |  |  |

# Supplementary Table 3. Summary of item revisions in the ADIMS questionnaire across the three waves of cognitive interviews

| Original version | Recommended changes and rationale (Wave 1 to 2) | Wave 2 version | Recommended changes and rationale (Wave 2 to 3) | Wave 3 Version | Recommended changes and rationale (Wave 3 to final) |
| --- | --- | --- | --- | --- | --- |
| Instructions  **Over the past 7 days, how short of breath did you get with each of these activities?** | Per feedback on recall period, this root question was added to each item in this PRO to remind participants of the correct recall period. With the ePRO, respondents will only see 1 item per screen. | Instruction applied at start of each item. | Add introductory screen to remind participants of recall period. | The next questions ask about your shortness of breath while doing various activities in the PAST 7 DAYS. *[Final 4 interviews only:]* If you avoided an activity **because of your shortness of breath**, please check “I was unable to do…” If you did not perform an activity for reasons **unrelated to heart failure**, please check “I did not have the opportunity…” | The first sentence is useful in informing respondents of the recall period. The remainder of these instructions are no longer necessary with the recommended revisions to the items. |
| Walking 10 steps/paces on flat ground at a normal speed without stopping | Given that a number of participants did not use the correct recall period, “over the past 7 days” was added to the item. Additionally, per comments by one participant regarding differences between “steps” and “paces” item was revised.  Bolded response period and “walking 10 steps…” part to emphasize recall period and physical task. | Over the **past 7 days**, how short of breath did you get while **walking 10 steps on flat ground without stopping**? | In parallel to wave 2 interviews, the linguistic team noted the difficulty of translating “a normal speed” and suggested a modified wording. Thus, “usual pace” replaced “normal speed.” | Over the **past 7 days**, how short of breath did you get while **walking 10 steps on flat ground at your usual pace without stopping**?” | Due to challenges in interpreting the response scale as intended, this item will be divided into items with two different response scales.  Additionally, “without stopping” wording will be removed because about a third of participants across all 3 waves included stopping to take breaks in their interpretation of difficulties performing mobility activities. |
| Walking a block (100 m) on flat ground at a normal speed without stopping | To be consistent with the previous item changes, “over the past 7 days” was added to the item. Bolded response period and “walking a block (10 0m)…” part to also be consistent with previous item. | Over the **past 7 days**, how short of breath did you get while **walking a block (100 m) on flat ground at a normal speed without stopping**? | In parallel to wave 2 interviews, the linguistic team noted the difficulty of translating “a normal speed” and suggested a modified wording. Thus, “usual pace” replaced “normal speed.” | Over the **past 7 days**, how short of breath did you get while **walking a block (100 meters) on flat ground at your usual pace without stopping**? | Due to challenges in interpreting the response scale as intended, this item will be divided into items with two different response scales.  Additionally, “without stopping” wording will be removed because about a third of participants across all 3 waves included stopping to take breaks in their interpretation of difficulties performing mobility activities. |
| Walking up 10 stairs (1 flight) without stopping | To be consistent with the previous item changes, “over the past 7 days” was added to the item. Bolded response period and “walking up 10 stairs (1 flight)…” part to also be consistent with previous item. | Over the **past 7 days**, how short of breath did you get while **walking up 10 stairs (1 flight) without stopping**? | No changes | Over the **past 7 days**, how short of breath did you get while **walking up 10 stairs (1 flight) without stopping**? | Due to challenges in interpreting the response scale as intended, this item will be divided into items with two different response scales.  Additionally, “without stopping” wording will be removed because about a third of participants across all 3 waves included stopping to take breaks in their interpretation of difficulties performing mobility activities. |
| Walking up an incline or hill at a normal speed without stopping | To be consistent with the previous item changes, “over the past 7 days” was added to the item. Bolded response period and “walking up an incline or hill…” part to also be consistent with previous item. | Over the **past 7 days**, how short of breath did you get while **walking up an incline or hill at a normal speed without stopping**? | The linguistic team noted the difficulty of translating “a normal speed” and suggested a modified wording. Added “usual pace” to item. | Over the **past 7 days**, how short of breath did you get while **walking up an incline or hill at your usual pace without stopping**? | Due to challenges in interpreting the response scale as intended, this item will be divided into items with two different response scales.  Additionally, “without stopping” wording will be removed because about a third of participants across all 3 waves included stopping to take breaks in their interpretation of difficulties performing mobility activities. |
| **Mobility domain response scale**  No shortness of breath  Mildly short of breath  Moderately short of breath  Severely short of breath  I was unable to do this because of shortness of breath  I did not do this in the past 7 days | No changes to the wording. The highlighting of the latter two options was modified so that the “I did not do…” was visually differentiated from “Unable to do.” | No shortness of breath  Mildly short of breath  Moderately short of breath  Severely short of breath  I was unable to do this because of shortness of breath  I did not do this in the past 7 days | “Did not do” option was intended to be equivalent to “not applicable” (i.e., did not have opportunity to do) but was interpreted as the most severe option by some (i.e., did not to because of symptom or possibility of worsening symptom). Revised last option to include “did not have the opportunity…” | No shortness of breath  Mildly short of breath  Moderately short of breath  Severely short of breath  I was unable to do this because of shortness of breath  I did not have the opportunity to do this in the past 7 days | Due to difficulties interpreting the scale, each mobility item was divided into two questions. |
| In the past 7 days, I had difficulty falling asleep **due to shortness of breath** … | No changes to wording but bolded response period for consistency. | In the **past 7 days**, I had difficulty falling asleep **due to shortness of breath** … | No changes | In the **past 7 days**, I had difficulty falling asleep **due to shortness of breath** … | No changes recommended |
| In the past 7 days, I had trouble staying asleep **due to shortness of breath** … | No changes to wording but bolded response period for consistency | In the **past 7 days**, I had trouble staying asleep **due to shortness of breath** … | No changes | In the **past 7 days**, I had trouble staying asleep **due to shortness of breath** … | No changes recommended |
| In the past 7 days, **shortness of breath** affected the quality of my sleep… | No changes to wording but bolded response period for consistency | In the **past 7 days, shortness of breath** affected the quality of my sleep… | No changes | In the **past 7 days, shortness of breath** affected the quality of my sleep… | No changes recommended |
| **Sleep domain response scale**  Never  Rarely  Sometimes  Often  Always | No changes prior to wave 2, however results from this wave were considered together with results from wave 2 to revise the scale prior to wave 3 | Never  Rarely  Sometimes  Often  Always | For more consistent interpretations of the scale, response options based on number of nights per week are recommended. ICON has done this successfully for similar concepts for another condition-specific PRO).  Revised sleep impact frequency response scale | **Sleep domain response scale**  Never  1-2 nights  3-4 nights  5-6 nights  Every night | No changes recommended |

# Supplementary Table 4. Summary of item revisions in the HF-FSA across the three waves of cognitive interviews

| Original Version | Recommended changes and rationale | Wave 2 Version | Recommended changes and rationale | Wave 3 Version | Recommended changes and rationale |
| --- | --- | --- | --- | --- | --- |
|  |  | *(No instructions for this PRO)* | At least one participant (see Item 1) used “yesterday” as a time frame instead of past 7 days. Add introductory screen stating. | **Instructions**  The next questions ask about your health status in general. Please think about the PAST 7 DAYS when answering. | No changes |
| Are you able to carry two bags filled with groceries? | Added recall period of 7 days and bolded activity to be consistent with previous set of items. Wording modified slightly to include phrase “how difficult was it”. | In the **past 7 days**, were you able to carry how difficult was it for you to **carry two bags filled with groceries**? | No changes | In the **past 7 days**, how difficult was it for you to **carry two bags filled with groceries**? | This item is redundant with the moderate-effort housework item, which also mentions “carrying groceries.” Therefore, this item can be removed. |
| Are you able to go for a walk for at least 15 minutes on flat ground at a normal pace? | Added recall period of 7 days and bolded activity to be consistent with previous set of items. Wording modified slightly to include phrase “how difficult was it”. | In the **past 7 days**, how difficult was it **to go for a walk for at least 15 minutes on flat ground at a normal pace**? | The linguistic team noted the difficulty of translating “a normal speed” and suggested a modified wording | In the **past 7 days**, how difficult was it **to go for a walk for at least 15 minutes on flat ground at your usual pace**? | Due to challenges in interpreting the response scale as intended, this item will be divided into items with two different response scales. |
| Are you able to wash and dry your body? | Added recall period of 7 days and bolded activity to be consistent with previous set of items. Wording modified slightly to include phrase “how difficult was it”. | In the **past 7 days**, how difficult was it for you to **wash and dry your body**? | No changes | In the **past 7 days**, how difficult was it for you to **wash and dry your body**? | Due to challenges in interpreting the response scale as intended, this item will be divided into items with two different response scales. The phrase “without any help” is added to clarify the meaning for basic activities, washing and dressing. |
| Are you able to dress yourself, including tying shoelaces and buttoning up your clothes? | Added recall period of 7 days and bolded activity to be consistent with previous set of items. Wording modified slightly to include phrase “how difficult was it”. | In the **past 7 days**, how difficult was it for you to **dress yourself**, including tying shoelaces and buttoning up your clothes? | No changes | In the **past 7 days**, how difficult was it for you to **dress yourself**, including tying shoelaces and buttoning up your clothes? | Due to challenges in interpreting the response scale as intended, this item will be divided into items with two different response scales. The phrase “without any help” is added to clarify the meaning for basic activities, washing and dressing. |
| Are you able to run errands and shop? | Added recall period of 7 days and bolded activity to be consistent with previous set of items. Wording modified slightly to include phrase “how difficult was it”. | In the **past 7 days**, how difficult was it for you to **run errands and shop**? | No changes | In the **past 7 days**, how difficult was it for you to **run errands and shop**? | Due to challenges in interpreting the response scale as intended, this item will be divided into items with two different response scales. |
| Does your health now limit you in going outside the home, for example to shop or visit a doctor’s office? | Removed this item, as it was redundant with the previous item “run errands and shop” |  |  |  |  |
| Does your health now limit you in doing moderate work around the house like vacuuming, sweeping floors or carrying in groceries? | Added recall period of 7 days and bolded activity to be consistent with previous set of items. Wording modified slightly to include phrase “how difficult was it”. | In the **past 7 days**, how difficult was it for you to do **moderate work around the house**, like vacuuming, sweeping floors, or carrying groceries? | The linguistic team recommended a slightly different wording for clarity across multiple languages. | In the **past 7 days**, how difficult was it for you to do **housework requiring moderate effort**, like vacuuming, sweeping floors, or carrying groceries? | Due to challenges in interpreting the response scale as intended, this item will be divided into items with two different response scales. |
| No item | Two patients reported that more rigorous activities, such as shovelling snow, were not included among impacts. Given this, an item about “heavy work” was added. | In the **past 7 days**, how difficult was it to do **heavy work around the house**, like scrubbing floors, or lifting or moving heavy furniture? | The linguistic team recommended a slightly different wording for clarity across multiple languages. | In the **past 7 days**, how difficult was it to do **housework requiring heavy effort**, like scrubbing floors, or lifting or moving heavy furniture? | Due to challenges in interpreting the response scale as intended, this item will be divided into items with two different response scales. |
| **Daily activities domain response scale:**  Without any difficulty  With a little difficulty  With some difficulty  With much difficulty  Unable to do | Given that the items were revised to included “how difficult was it”, the response options were revised accordingly. Also, added “I did not do this in the past 7 days” to be consistent with changes on the SOB measure. | **Daily activities domain response scale**  Not difficult  A little difficult  Somewhat difficult  Very difficult  Unable to do  I did not have the opportunity to do this in the past 7 days | The linguistic team noted the ambiguity between “a little” and “somewhat” difficult and recommended “moderately” to replace somewhat. | **Daily activities domain response scale**  Not difficult  A little difficult  Moderately difficult  Very difficult  Unable to do  I did not have the opportunity to do this in the past 7 days | Due to challenges in interpreting the response scale as intended, this item will be divided into items with two different response scales. |
| In the past 7 days, I have been able to concentrate…  … | Added recall period of 7 days and bolded activity to be consistent with previous set of items. | In the **past 7 days**, I have been **able to concentrate**… | No changes | In the **past 7 days**, I have been **able to concentrate**… | No changes |
| In the past 7 days, I have been able to remember things as easily as usual without extra effort… | Added recall period of 7 days and bolded activity to be consistent with previous set of items. | In the **past 7 days**, I have been able to **remember things as easily as usual** without extra effort… | Due to confusion with the response scale, the wording of this item was simplified. | In the **past 7 days**, I have been able to **remember things**… | No changes |
| **Cognitive ability domain response scale**  Not at all  A little bit  Somewhat  Quite a bit  Very much | No changes | Not at all  A little bit  Somewhat  Quite a bit  Very much | The scale was reversed so that the direction was consistent with the daily activities domain response scale; wording of options was revised according to the new item wording. | **Cognitive ability domain response scale**  *For ability to concentrate item:*  With no difficulty  With a little difficulty  With moderate difficulty  With great difficulty  Not at all  *For ability to remember things item:*  With no difficulty (as usual)  With a little difficulty  With moderate difficulty  With great difficulty  Not at all | Recommend removing the final option “not at all,” as this is the only one causing confusion, and would likely not be relevant to someone who is able to self-complete a questionnaire. |

**Supplementary Figure 1. PRISMA diagram for targeted literature review**


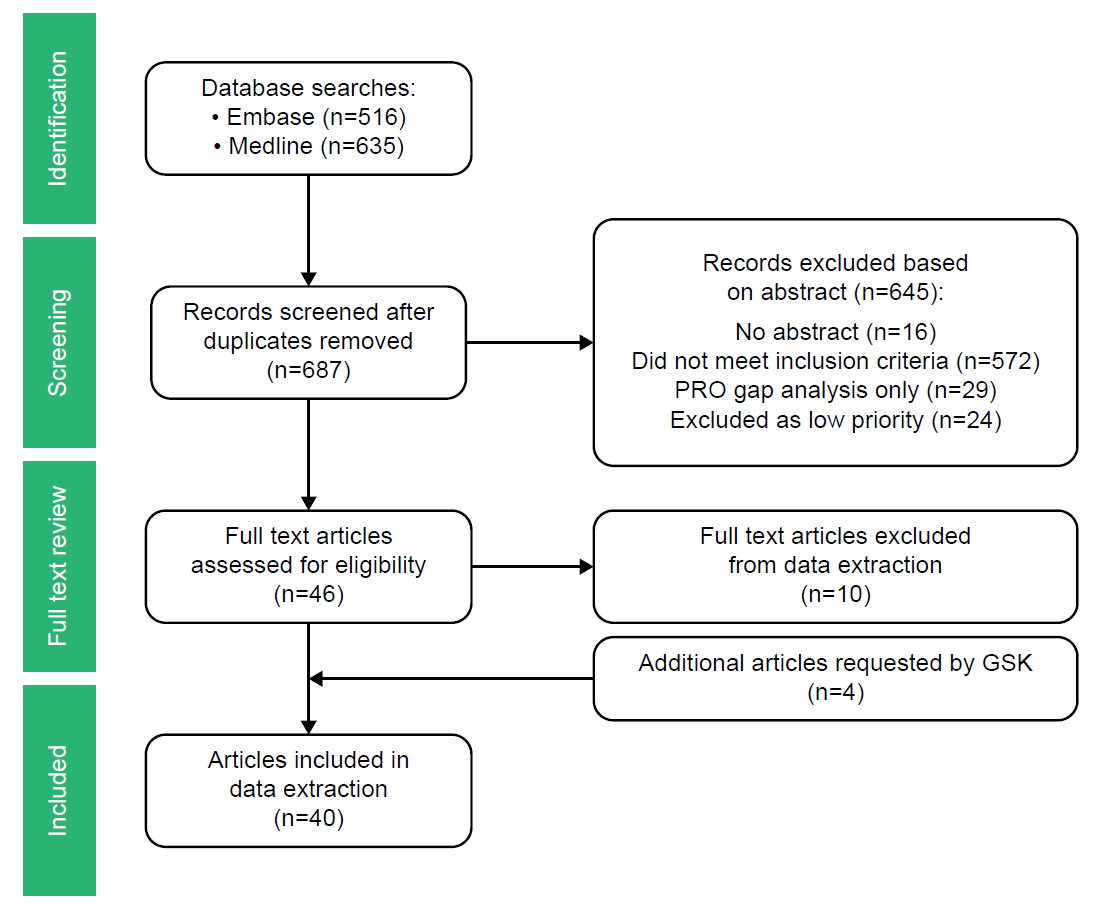


GSK, GlaxoSmithKline; PRISMA, Preferred Reporting Items for Systematic Reviews and Meta-Analyses; PRO, patient-reported outcome.
